# Planetary Health Diet for Childhood Obesity Prevention: Integrating Nutritional Health with Environmental Stewardship

**DOI:** 10.3390/nu16244316

**Published:** 2024-12-13

**Authors:** Maria Vittoria Conti, Alessandra Vincenti, Alice Beretta, Valeria Calcaterra, Silvia Taranto, Marianna Diotti, Gianvincenzo Zuccotti, Hellas Cena

**Affiliations:** 1 Laboratory of Dietetics and Clinical Nutrition, Department of Public Health, Experimental and Forensic Medicine, University of Pavia, 27100 Pavia, Italy; mariavittoria.conti@unipv.it (M.V.C.); alice.beretta@unipv.it (A.B.); hellas.cena@unipv.it (H.C.); 2 Pediatric and Adolescent Unit, Department of Internal Medicine, University of Pavia, 27100 Pavia, Italy; valeria.calcaterra@unipv.it; 3 Pediatric Department, Buzzi Children’s Hospital, 20154 Milano, Italymarianna.diotti@gmail.com (M.D.); gianvincenzo.zuccotti@unimi.it (G.Z.); 4 Department of Biomedical and Clinical Science, University of Milan, 20157 Milano, Italy; 5 Clinical Nutrition and Dietetics Service, Unit of Internal Medicine and Endocrinology, Istituti Clinici Scientifici Maugeri IRCCS, 27100 Pavia, Italy

**Keywords:** obesity, children, planetary health diet, EAT-Lancet Commission, sustainable diet

## Abstract

Background: Childhood obesity is a critical public health challenge with a rising prevalence worldwide, contributing to numerous health risks and long-term societal burdens. Concurrently, climate change and environmental degradation demand sustainable approaches to dietary patterns. The Planetary Health Diet (PHD), initially designed for adults, emphasizes plant-based foods and sustainable practices. Objectives: This review explores the suitability of the PHD for addressing childhood obesity by assessing its nutritional adequacy and proposing necessary adaptations for pediatric populations. Methods: A narrative review methodology was employed, examining data from global and regional nutritional guidelines and evaluating the PHD’s bromatological composition against age-specific requirements. Results: The findings indicate that while the PHD aligns with environmental goals, it may not fully meet the energy and specific nutrient needs of children and adolescents without modifications. Key challenges include ensuring adequate intake of bioavailable protein, iron, calcium, vitamin B12, and vitamin D. Strategies such as incorporating fortified foods, optimizing food pairings, and gradual adaptation to high-fiber diets are critical for successful implementation. This review also highlights the importance of cultural adaptability, family involvement, and regional food systems in promoting adherence. Tailored interventions, such as school meal programs and educational initiatives, can bridge gaps in nutrition while fostering sustainable dietary behaviors. Conclusions: Adapting the PHD for pediatric needs presents an opportunity to integrate nutritional health with environmental stewardship, contributing to obesity prevention and a sustainable future. Further research is required to validate these adaptations and develop comprehensive frameworks for global implementation.

## 1. Introduction

Childhood obesity is a growing global health concern, with over 38 million children under five being overweight in 2019, particularly in low- and middle-income countries [1]. This increases the risk of type 2 diabetes, cardiovascular disease, psychological issues, and adult obesity [2]. Simultaneously, malnutrition in all its forms (i.e., obesity, undernutrition, and other dietary inadequacies) remains the leading global health risk, compounded by climate change, which exacerbates vulnerabilities [3]. Together, obesity, undernutrition, and climate change form a “Global Syndemic”, where intertwined pandemics amplify one another and share societal drivers. Tackling childhood obesity within this context is imperative to ensure both human and planetary health [4].

The EAT-Lancet Commission’s Planetary Health Diet (PHD) serves as a universal guideline to promote human health and environmental sustainability [5]. It emphasizes predominantly plant-based foods while reducing animal-sourced foods and limiting processed and red meats [5]. While designed for adults, there is growing interest in its potential for children, given its alignment with health and environmental goals [5].

Classic approaches to combating childhood obesity often focus on caloric restriction or limiting specific macronutrients, which can be difficult to maintain over time. Standardized diets, which are not always adaptable to different cultures or local contexts and individual interventions, may be less effective compared to integrated strategies. The PHD, on the other hand, offers a holistic and modern approach, simultaneously addressing children’s health, development, and planetary sustainability.

Children’s unique nutritional needs, driven by rapid growth and development, differ significantly from those of adults, requiring higher intakes of specific macronutrients and micronutrients [6]. Strict adherence to the adult PHD, without modifications, raises concerns about potential nutrient deficiencies or inadequate energy intake, which could hinder healthy development [7]. Additionally, the complexity of defining a “healthy” diet for children varies across age groups and cultural contexts [8], highlighting the need for tailored approaches. Research underscores the importance of tailored dietary guidelines for children, particularly for managing metabolic conditions such as obesity and related complications [9]. These interventions consider not only caloric and nutritional content but also food quality, which is critical to preventing and managing childhood obesity [9].

This review evaluates the suitability of the PHD for children, examining its nutritional adequacy, necessary adaptations to meet pediatric requirements, and its potential role in addressing childhood obesity while advancing environmental sustainability. By bridging gaps in research, it seeks to provide a framework for integrating sustainable diets into children’s nutrition, addressing the dual challenges of public health and environmental sustainability.

To further contextualize the adoption of the PHD for pediatric populations, it is important to consider existing dietary guidelines and interventions targeting childhood obesity. Current guidelines, such as those from the World Health Organization (WHO), emphasize nutrient-rich, balanced diets that address both caloric needs and micronutrient deficiencies in children. Integrating these established principles with the sustainability focus of the PHD could create a holistic approach to tackling obesity while promoting environmental stewardship.

## 2. Materials and Methods

A narrative review search strategy was used to identify relevant research on the suitability of the PHD for children’s nutritional requirements, with a specific focus on its potential impact on counteracting childhood obesity. An extensive literature search on PubMed was conducted, with the language being restricted to English. The most pertinent original scientific papers, clinical trials, meta-analyses, and systematic reviews were included, and case reports, case series, brief reports, commentaries, editorials, and gray literature sources were excluded due to the lack of generalizability, low level of evidence, and methodological limitations.

The research terms adopted were “children” and “childhood obesity” or “pediatric obesity” or “obesity” or “overweight” and “Planetary Diet” or “Planetary Healthy Diet” or “Eat Lancet Diet”.

In order to compare whether the PHD model meets the needs of an adult subject (in terms of energy and macro- and micronutrients), the following methodology was adopted: referring to national and international organizations/guidelines, the nutrient requirements for the adult population were identified from the latest version of the “Reference Intake Levels for Nutrients and Energy” 2024 [10] (national level), from the EFSA [11,12,13,14,15,16,17,18,19,20,21,22,23,24,25,26,27,28,29,30,31] (European level), and finally from the FAO and WHO [32,33,34,35,36,37,38,39,40] (international level).

It should be specified that the reference values that were taken into consideration were the nutrient requirements for an adult of average height and weight physiological condition.

Both average requirements for macronutrients such as water, complex carbohydrates, simple sugars, fiber, total lipids, saturated fat, PUFA, PUFA-omega 3, cholesterol, and protein and micronutrients such as vitamin B6, vitamin B9, vitamin B12, vitamin C, vitamin A, vitamin D, vitamin E, sodium, potassium, magnesium, calcium, phosphorus, iron, zinc, iodine, and selenium were evaluated.

Where specified, differences in requirements between male and female adult subjects were highlighted.

Table 1 specifies the reference values for adequate intake (AI), acceptable macronutrient distribution range (AMDR), Suggested Dietary Intake (SDT), reference intake range for macronutrients (RI), average requirement (AR), population reference intake (PRI), and tolerable Upper intake Level (UL).

The assessment of the bromatology composition of the PHD model was calculated using the META Diet software, 2016 version (https://www.metadieta.it/software/ accessed on 10 November 2024). The value for the various food categories proposed by the (PHD) was used as reported:

- Rice, wheat, corn, and other: 232 g (specific value);
- Potatoes and cassava: 50 g (average value of the reference range);
- Vegetables: 300 g (average value of the reference range);
- Fruits: 200 g (average value of the reference range);
- Dairy foods: 200 g for milk plus 50 g derivative equivalents (average value of the reference range);
- Beef/lamb/pork: 14 g (specific value);
- Chicken/other white meats: 29 g (average value of the reference range);
- Eggs: 18 g (average value of the reference range);
- Fish: 28 g (average value of the reference range);
- Legumes: 75 g (average value of the reference range);
- Nuts: 50 g (average value of the reference range);
- Unsaturated fats: 40 g (average value of the reference range);
- Saturated fats: 11.8 g (maximum value);
- Simple sugars: 31 g (maximum value).

The bromatology assessment was calculated based on a typical day by entering the specific quantities for the individual food categories. Where available in the software, the “average” option was chosen for the composition of different food groups, such as vegetables, fruit, hard cheeses, fish, pulses, and dried fruit.

To evaluate the adequacy of the PHD for children and adolescents’ macro- and micronutrient requirements, the LARN [10], EFSA [11,12,13,14,15,16,17,18,19,20,21,22,23,24,25,26,27,28,29,30,31], and FAO/WHO [32,33,34,35,36,37,38,39,40] recommendations were used. The evaluation was carried out using the following methodology: the bromatological composition of the PHD, calculated from adult portions, was adjusted according to the energy recommended as adequate by the three authorities for the different age groups and for each sex. Then, the values obtained for energy, protein, fiber, minerals, and vitamins were compared with the recommendations of the three main authorities considered. The requirement was assessed as adequate (reported in green color) if the dietary intake was equal to or greater than the recommendations and relatively adequate (reported in yellow color) if the intake was more than 5 percent below the target value.

The energy, protein, lipid (total, SFA, AG trans, MUFA, PUFA, PUFA *n*-6, and PUFA *n*-3), carbohydrate (total, sugar, dietary fiber), vitamin (B6, B9, B12, C, A, E, and D), and mineral (sodium, potassium, magnesium, calcium, phosphorus, iron, zinc, iodine, and selenium) requirements for children and adolescents aged 2 to 17 years as defined by the three main authorities, as well as the evaluation of the PHD’s adequacy according to those recommendations, are reported in the Supplementary Materials.

## 3. Results

### 3.1. Nutritional Requirements of Children Compared to Adults

The PHD stems from the EAT-Lancet Commission report, which was developed to address the dual challenge of providing nutritious food to a growing population while reducing environmental degradation [5]. It is designed to promote human health and ensure environmental sustainability by emphasizing the consumption of plant-based foods, such as fruits, vegetables, legumes, wholegrains, and nuts, while reducing the intake of animal-based foods, particularly red and processed meats [5]. This model also focuses on reducing food waste and optimizing land and water use to prevent further harm to ecosystems. It aligns with the principles of sustainable development and climate change mitigation by encouraging the production and consumption of foods with lower environmental footprints, such as those with reduced greenhouse gas emissions, minimal use of water and land resources, and low biodiversity impacts [5,41].

While the PHD aligns with various traditional eating patterns, it does not require the global population to eat the same foods or adhere to a specific diet. Instead, it outlines general food categories and intake ranges that could enhance human health when incorporated into a diet. It is crucial to interpret and adapt the universal principles of the PHD to reflect local cultures, geographies, and demographics [41].

The PHD model has been designed and developed to meet the needs of a healthy adult of average weight and height, based on global population parameters. However, given its positive impact on human health [41,42,43], this model can also be applied to help the prevention of childhood obesity. In this context, the PHD needs to be adapted to meet specific energy and macro- and micronutrient requirements for growth and development, while still maintaining its environmental focus.

Taking the PHD model into consideration, the authors evaluated whether it can meet the needs of an average adult (Table 1), both in terms of macro- and micronutrients, and then assessed how to adapt this model to a population of developmental-age subjects (Annex 1). The comparison of the bromatological composition of the PHD model (more on how it was evaluated is specified in the Section 2) with reference levels of energy and nutrients for an adult subject (referring to LARN [10]; EFSA [11,12,13,14,15,16,17,18,19,20,21,22,23,24,25,26,27,28,29,30,31]; and WHO [32,33,34,35,36,37,38,39,40]) shows good consistency (Table 1).

### 3.2. Assessing the Adequacy of the Planetary Health Diet for Children’s Health and Energy Needs

Childhood is a critical period for growth, development, and the establishment of healthy eating patterns that can prevent obesity and promote long-term health. Understanding the specific nutritional needs of children is essential for effective dietary recommendations, especially within the framework of the PHD, which emphasizes both nutritional health and environmental sustainability.

The following will briefly describe the adequacy of the bromatological composition of a typical day that includes all the food groups indicated by the PHD, in the quantities suggested and reported in the Materials and Methods section, compared with the sex- and age-specific recommendations given by the LARN, EFSA, and FAO/WHO. The recommendations are reported in the Supplementary Materials (Tables S1–S18).

The PHD provides on average 2500 kcal per day. Considering a Physical Activity Level (PAL) equal to 1.6, female energy requirements from 2 to 18 years of age are fully satisfied according to the LARN and EFSA, but only until 12 years of age according to the WHO/FAO. On the contrary, according to all the three main authorities, the PHD satisfies the energy needs of males only up to 12 years of age (Supplementary Materials—Table S25a). Indeed, for males from 13–14 years of age up to their 18th birthday, their energy needs vary from a minimum of 2508 to a maximum of 3400, thus requiring a higher intake than that stipulated by the PHD and therefore potentially going beyond sustainable boundaries (Supplementary Materials—Table S25a). Moreover, for extremely active children and adolescents (PAL: 1.8–2.0), their requirements also reach values between 3400 and 4000 kcal/day. However, it should be remembered that this represents a particular population group, which therefore requires a specific and personalized assessment of its needs.

Daily dietary fiber and protein requirements are fully met with the PHD for every energy requirement (Supplementary Materials—Table S25b,c). Regarding micronutrients, the requirements, as defined by the three main authorities, for vitamins B6, B9, B12, C, A, and E are fully met when consuming a complete PHD, with all food groups, even when adjusting for the energy requirements of different age groups (Supplementary Materials—Table S25d–i). However, the PHD, like all other dietary patterns, does not allow the nutritional requirement for vitamin D to be met (Supplementary Materials—Table S25l). By consuming a diet based on the PHD, sodium, calcium, and iodine requirements are not met, even when adjusting for energy requirements, for both males and females, in all age categories (Supplementary Materials—Table S25m,p,t). On the contrary, magnesium, phosphorus, and zinc requirements are fully met when consuming a PHD, even when adjusting for energy requirements, according to all three main authorities’ recommendations (Supplementary Materials—Table S25q,o,s). The iron requirement is adequately satisfied in almost all age categories in males, but it is completely inadequate in satisfying the needs of females of a fertile age (Supplementary Materials—Table S25r). The PHD does not guarantee the satisfaction of age-specific selenium requirements as defined by the LARN and EFSA, but it does meet the requirements defined by the WHO (Supplementary Materials—Table S25u), while for potassium, the intake is below the Italian recommendations issued by the LARN, but appears adequate for all age categories compared to the needs defined by the EFSA (Supplementary Materials—Table S25n). The complete analysis is reported in the Supplementary Materials.

### 3.3. Adapting the Planetary Health Diet for Pediatric Nutrition

Adapting the PHD for pediatric nutrition requires careful consideration to meet children’s unique growth needs while aligning with the principles of sustainability, given that individuals in this population are in critical phases of development [43,44].

The PHD can be adapted to meet the protein needs of children and adolescents (due to their rapid growth, physical development, and active lifestyles) [10,14,33], but this requires careful planning to ensure that their specific nutritional requirements are fulfilled. The PHD’s primary reliance on plant-based proteins such as legumes (e.g., beans, lentils, peas, chickpeas) can provide a significant amount of proteins [5]. In addition to plant-based proteins, the PHD can be adjusted to include moderate amounts of high-quality animal proteins such as eggs, dairy, lean meats, and fish that offer all the essential amino acids in a highly bioavailable form. In addition, fortified plant-based foods, such as soy products and plant milks that are enriched with protein and essential vitamins, can also be effective in supplementing the diet. This ensures that nutrient needs, including amino acids and micronutrients such as iron and vitamin B12, are met.

Fruits, vegetables, and wholegrains (including rice, wheat, corn, and other cereals) are core components of the PHD and should be consumed in generous amounts for their fiber, in addition to their vitamins and minerals [5]. Introducing and consuming fiber for children and adolescents is essential for promoting their gut and digestive health, supporting metabolic function and maintaining balanced energy levels [45]. The importance of fiber intake in children and adults is likely based on the chemical structure of fiber, which varies in chain length, branching, side chains, type of binding, and composition, all of which may alter the function in the human gut and have effects on disease [45]. However, many foods based on high-fiber grains are not well accepted by children, and therefore, a gradual approach is recommended. Sudden increases can cause digestive issues like bloating or discomfort, particularly if a child or adolescent is not used to a fiber-rich diet. Starting with small amounts and gradually increasing them over time allows their digestive system to adjust.

Meeting the vitamin and mineral needs of children and adolescents while following the PHD requires a focused approach. Concerning iron intake, legumes, lentils, spinach, fortified cereals, and wholegrains are the main iron (non-heme) sources in the PHD. Since this form of iron is less bioavailable than heme iron from animal products, it is important to enhance its absorption by pairing these foods with vitamin-C-rich options (e.g., oranges, strawberries, or peppers).

Calcium and vitamin D are vital for bone health, especially during periods of rapid growth [46]. To adapt the PHD, plant-based calcium sources like fortified plant milks (such as almond, soy, or oat milk), almonds, chia seeds, and leafy greens (e.g., kale and bok choy) should be included. Given the limited natural food sources of vitamin D, especially in a diet that restricts animal products, fortified foods and responsible sun exposure are essential. In some cases, supplementation might be necessary, particularly in regions with low sunlight.

Vitamin12 is primarily found in animal-based foods (i.e., meat, dairy, eggs, and fish). Since the PHD emphasizes plant-based foods and reduces the consumption of animal products, it presents a unique challenge in ensuring that individuals, especially children and adolescents, meet their vitamin B12 requirements. The primary challenge with the PHD is that vitamin B12 is not naturally present in plant foods, except in trace amounts found in certain mushrooms or fermented foods, which are generally not enough to meet the daily requirement [47]. Therefore, individuals following the PHD need to rely on fortified foods or supplements to ensure they are getting sufficient vitamin B12. Fortified foods are an essential part of the PHD, as many plant-based foods, such as non-dairy milks (e.g., soy, almond, oat), breakfast cereals, and plant-based meat alternatives, are commonly fortified with vitamin B12. These fortified products help bridge the gap in B12 intake for those avoiding animal products. Regular monitoring of vitamin B12 levels helps avoid deficiencies.

Besides quantitative and qualitative adaptation of the PHD for children, the authors recognize that the household plays a crucial role in shaping and adapting a diet for children and adolescents, as the home environment is where eating habits are formed [48,49]. Specifically, caregivers are often gatekeepers for the diets of infants and children. They are responsible for procuring and preparing foods for and supervising the eating practices of young children and are therefore potentially responsible for modeling dietary behaviors [50]. The ability of children to emulate the actions of others and learn through observation, especially from parents or caregivers, may explain different types of dietary styles [51]. The family is a system where a positive environment can be created that establishes and promotes beneficial health behaviors through role modeling, the provision of “healthy” food, and support for engagement in healthy eating behaviors [50,52]. The family’s role in fostering children’s adaptation to the PHD is pivotal. Through positive role modeling, creating an encouraging home environment, effective communication, and shared experiences, families can nurture children’s openness to dietary practices that are both healthful and sustainable.

While the PHD recommendations should aim to balance nutrition, health, accessibility, and environmental purposes, to properly and efficiently reach their goal, they should be adapted to specific local and national contexts [44,53]. Indeed, local food cultures, traditions, and norms play an important role in shaping the dietary practices of a nation [54]. Therefore, the transferability and adoption of the PHD across all nations could present a number of challenges [55]. Food insecurity and barriers to adoption due to religion, culture, traditional cooking practices, and the economic burden need to be addressed in interventions aiming to increase adaptation to the PHD for pediatric nutrition. Incorporating foods that are culturally acceptable, locally produced, and accessible, but with a similar nutritional profile to those proposed by the PHD, may encourage adherence. Such dietary pattern adaptations which focus on locally produced and acceptable foods have been studied for other dietary patterns such as the Mediterranean diet (MD) [56,57]. George et al. developed an MD model complying with the principles of the traditional MD and applied it in two clinical trials in a multiethnic context in Australia [57]. Firstly, the nutrient profile of the MD was identified by the authors from previous MD interventions, and this was then used to identify key food-based components, with these two steps being combined to develop a 2-week meal plan. Potential barriers (i.e., adaptability to cultural preferences) to translating the MD into the Australian population habits were then identified, with appropriate strategies being embedded into the intervention (cultural dishes) to address these perceived barriers [57]. Regional and cultural adaptations are also important from an environmental and cost perspective due to their alignment with local resources and ecosystems [5,58].

Additionally, crops that are native to a particular region usually require fewer resources, such as water and pesticides, because they are already well suited to the local climate, soil, and weather conditions [5]. This natural alignment conserves resources and minimizes the environmental impact of farming. Furthermore, by focusing on traditional, local crops, regional ecosystems and biodiversity are better preserved, supporting local wildlife and maintaining a balanced ecosystem [59]. Overall, regional adaptations help mitigate the environmental impact, support local economies, and enhance public health [59].

### 3.4. Impact on Counteracting Childhood Obesity

A healthy diet is key to preventing childhood obesity, with the PHD offering a dual benefit by promoting health and environmental sustainability. Poor dietary habits, including excessive consumption of ultra-processed foods and sugar-sweetened beverages (SSBs) are significant contributors to obesity worldwide [60,61,62,63]. SSBs, in particular, provide liquid calories that fail to promote satiety, leading to excessive energy intake. Overconsumption of fructose from SSBs also disrupts metabolic health, contributing to fat deposition, insulin resistance, and increased cardiovascular risk [64]. Overconsumption of fructose can result in increased hepatic de novo lipogenesis, visceral fat deposition, dyslipidemia, IR, and hepatic uric acid, which can cause hyperuricemia and consequently increase the risk of cardiovascular disease, DMT2, metabolic syndrome, and hypertension. Excessive SSB consumption also damages the gut microbiome by causing dysbiosis, which increases the risk of chronic diseases [64].

Ultra-processed foods are a staple in many children’s diets, especially at school, due to their convenience and taste. However, these foods often contain endocrine disruptors that interfere with hormonal regulation, promoting obesity through increased fat storage, reduced metabolism, and gut microbiota inefficiency. Additionally, their environmental impact is significant, with excessive packaging contributing to waste. Food processing and packaging are also common sources of ED, and, in particular, there is a positive association between bisphenol A, a plastic compound, and childhood obesity [65].

To reduce caloric intake from highly processed foods and sugary beverages, the PHD encourages a variety of plant-based foods that are typically rich in fiber, vitamins, and minerals. Plant-based diets benefit both health and the environment, making them a promising strategy to combat childhood obesity. Studies show that vegetarian children tend to have a lower BMI, particularly during adolescence [66,67]. These diets are rich in fruits, vegetables, and fiber, which promote satiety and reduce overall energy intake, helping prevent obesity.

Vegan and vegetarian children tend to consume more fruits and vegetables than their omnivorous peers, benefitting from these foods’ low energy density, high micronutrient content, and fiber. A high fiber intake, linked to satiety and reduced energy consumption, also supports metabolic health by lowering inflammatory markers. Meals that are high in carbohydrates can also raise the energy expenditure at rest. Male vegetarians, for instance, have a resting metabolic rate that is 11% higher than that of omnivores. Plant-based diets, rich in antioxidants and polyunsaturated fats, can improve glucose metabolism, lipid profiles, and resting metabolic rates, while reducing the consumption of calorie-dense animal products, which further aids weight management [66,68]. The gut microbiota can also benefit from a plant-based diet, which increases its ability to metabolize bile acids, choline, L-carnitine, amino acids, fiber, and polyphenols. Because meat has a high calorie density and is heavy in fatty acids, avoiding it can help an individual maintain a healthy weight. A high ratio of polyunsaturated to saturated fatty acids, which raises the resting metabolic rate by 3.6%, is a characteristic of a plant-based diet. With reduced blood lipid and cholesterol levels, a plant-based diet is linked to an improved lipid profile. Furthermore, some research has linked a high protein intake to a higher BMI and a higher chance of becoming obese in later life. Lastly, a plant-based diet may help enhance glucose metabolism, lower blood pressure, and lessen insulin resistance [66,68].

Integrating a vegan diet within the PHD framework offers a powerful strategy for preventing childhood obesity while promoting sustainability. According to current research, vegan children often exhibit lower weight, height, and fat mass than omnivores, whereas vegetarians show comparable or slightly lower measures [69]. For instance, the VeChi Diet Study reported fewer cases of overweight or obesity among vegan (18%) and vegetarian (18.1%) toddlers compared to omnivores (23.2%), although some vegan children were classified as stunted or wasted [69,70].

In a German publication that compared the anthropometric data of 430 toddlers between the ages of one and three who were vegetarian, vegan, or omnivorous, 23.2% of omnivorous children were categorized as overweight or at risk of obesity, compared to 18% of vegan children and 18.1% of vegetarian children. There were no differences in total energy intake between omnivorous and vegan children, but omnivorous children’s calorie intake was predominantly from protein, fat, and added sugars, while vegan children relied more on carbohydrates, including fiber. Compared to vegetarians (2.4–0%) and omnivores (0–0.6%), a greater proportion of vegan children (3.6–3.6%) were classified as stunted or wasted [70]. In the Vechi Youth Study, a sample of 149 vegetarian, 115 vegan, and 137 omnivorous children and adolescents (6–18 years old) had their anthropometry, dietary intake, and nutritional status evaluated. The height, body weight, and BMI were similar in all groups, but the vegan individuals had the lowest serum TC, LDL-C, and non-HDL-C concentrations, offering the first evidence that a vegan diet in itself can reduce cardiovascular risk factors in children and adolescents [71]. In a cross-sectional study examining Polish children aged 5–10 years, in a sample of 63 vegetarians, 52 vegans, and 72 comparable omnivores, the results showed that vegetarians had lower gluteofemoral adiposity but similar total fat mass and lean mass to omnivores, while vegans had lower fat indices in all regions and similar lean mass to their omnivorous counterparts [69].

However, longitudinal research, such as a Canadian cohort, noted a higher risk of underweight among vegetarian children, emphasizing the need for careful dietary planning [72].

Culture- and age-specific considerations also emerge. A Czech study found vegan children to be more likely to fall into lower BMI percentiles compared to omnivores, although the growth patterns remained consistent across groups [73,74]. Similarly, an Italian study of infants reported lower BMI and shorter body length among vegan children, but harmonious growth overall [74]. These findings underscore the potential of plant-based diets for health and environmental goals, provided that they are carefully monitored to ensure nutritional adequacy.

### 3.5. Case Studies and Practical Applications

The PHD encourages educational initiatives that teach children and families about healthy, sustainable eating. Schools and community programs can play a vital role in promoting awareness of the link between food choices, personal health, and the planet.

School meal programs play a pivotal role in shaping dietary habits during critical developmental years, offering a strategic opportunity to enhance both public health and sustainability. Several countries have implemented measures to reduce food-related greenhouse gas emissions while improving children’s nutrition. For example, Brazil’s School Feeding Program (PNAE) sources diverse, nutrient-dense foods locally, lowering obesity rates in low-income regions [75]. Studies indicate that schools with well-structured feeding programs like the PNAE have lower levels of overweight and obesity [76]. Japan integrates food education (Shokuiku) into school meal programs, using them as a “living textbook” to promote lifelong healthy eating, resulting in greater consumption of vegetables and protein-rich foods compared to children without school lunches [77]. Similarly, Sweden’s optimized school menus reduce emissions by 40%, meet nutritional standards, and cut costs by 11% showing that sustainable menus can also be cost-effective and appealing [78]. The EU’s School Scheme for Fruits, Vegetables, and Milk further demonstrates how such initiatives raise awareness and consumption of healthier foods among children [79].

The application of the PHD as a model for school meal programs is still in its early stages, with few studies assessing children and adolescents’ adherence. To address this, Cacau et al. developed the Planetary Health Diet Index (PHDI), demonstrating in a Brazilian cohort that higher adherence to the PHD correlated with a 24% lower likelihood of overweight or obesity [80,81]. However, adherence varies widely across populations. For instance, studies on Brazilian and Finnish children revealed low consumption of plant-based foods like legumes and nuts, coupled with high intakes of red meat and added sugars [82,83]. Mexican adolescents also exceeded the caloric recommendations for most food groups in the PHD framework [84]. To better measure adherence, Venegas Hargous et al. adapted the PHDI for children and adolescents (PHDI-C) [85], showing low adherence among Chilean preschoolers, particularly those with younger mothers.

While the correlations between PHD adherence and obesity in children remain inconclusive, Portuguese research found that a higher adherence to the PHD reduced obesity rates at age seven, emphasizing the importance of standardized metrics for evaluation [86,87,88].

Despite promising outcomes, the implementation of sustainable diets like the PHD faces challenges. Menus must account for regional food habits, cultural diversity, and dietary restrictions while being appealing to students [88]. The gradual introduction of plant-based meals and better seasoning and presentation can improve acceptance. Parent education, cooking classes, and taste tests have also proven effective in gaining support for dietary changes at home and in school [89].

Logistical issues such as sourcing local ingredients remain barriers. In Japan, the Ministry of Agriculture facilitates “local production for local consumption”, ensuring that schools can access affordable, high-quality local products despite cost and supply challenges. By 2021, 56% of school lunch ingredients in Japan were locally sourced [90]. Similarly, Israeli programs address budget constraints by working closely with the food industry and conducting frequent inspections to ensure quality [77].

Cultural adaptability is crucial for integrating the PHD into children’s diets globally. In Mediterranean countries, leveraging traditional eating patterns has proven effective, with adaptations like the MED_EAT-IT pattern balancing nutritional adequacy and environmental impact. While meeting most nutritional requirements except for vitamin D, this model aligns with local values and preferences [91]. Such culturally sensitive approaches enhance acceptance and reinforce the role of sustainable food systems in local communities.

## 4. Discussion

The Planetary Health Diet represents a transformative approach to addressing the dual challenges of childhood obesity and environmental degradation. This narrative review provides a detailed analysis of the PHD’s potential, emphasizing its alignment with sustainable dietary practices and its adaptability to pediatric populations. The findings suggest that while the PHD’s emphasis on plant-based, nutrient-dense foods supports obesity prevention and environmental stewardship, modifications are necessary to ensure its nutritional adequacy for children and adolescents.

Children and adolescents have specific nutritional needs, including higher energy or micronutrient requirements compared to adults. While the PHD meets the energy requirements of younger children, it falls short for adolescents, particularly males and highly active individuals. The gap between the caloric provisions of the PHD and the demands of growth and physical activity in adolescence underscores the need for tailored energy modifications to prevent nutritional deficiencies and ensure optimal development. Protein adequacy, a cornerstone of pediatric growth, poses a critical challenge within the PHD framework. Although plant-based proteins form the diet’s foundation, their bioavailability and amino acid profile differ from those of animal-based sources. Strategies such as incorporating fortified foods, leveraging complementary proteins, and including moderate amounts of high-quality animal proteins, such as dairy, eggs, and fish, are vital to bridge this gap. Similarly, the diet’s reliance on non-heme iron sources raises concerns about bioavailability, necessitating enhancements through vitamin-C-rich pairings or fortified products. Micronutrient adequacy remains a critical area for adaptation. For instance, calcium and vitamin D, essential for skeletal growth, are inadequately addressed by the PHD in its current form. Fortified plant-based milks, leafy greens, and responsible sun exposure, supplemented by vitamin D supplementation when necessary, are recommended to meet these demands. Vitamin B12 deficiency, an inherent limitation in plant-based diets, further underscores the need for fortified foods and regular monitoring. These adaptations are essential for aligning the PHD with the nutritional needs of children and adolescents. Another important aspect to consider is the influence of cultural and socioeconomic factors on the implementation of and adherence to the PHD. While the diet’s flexibility allows for regional and cultural adaptations, disparities in food access, traditional practices, and economic barriers may hinder its adoption. For example, the cost and availability of fortified foods or plant-based substitutes pose challenges in low- and middle-income settings. Additionally, palatability issues, especially regarding high-fiber foods, highlight the need for gradual dietary transitions and education to foster acceptance among children. In light of what has been said, there are encouraging data, and case studies of school meal programs demonstrate the PHD’s potential to drive systemic change [75,76,77,78,79]. Initiatives in Brazil, Japan, and Sweden show that integrating sustainable and nutritionally balanced meals in schools can improve dietary habits and promote health outcomes [75,76,77,78,79]. These examples underscore the importance of structured educational programs and community involvement in fostering long-term adherence. However, the variability in adherence rates, as observed in studies from Brazil, Finland, and Mexico, underscores the need for localized interventions that are tailored to dietary patterns and cultural contexts [75,76,77,78,79]. Moreover, the PHD offers significant potential for countering childhood obesity, primarily through its emphasis on reducing ultra-processed foods and sugar-sweetened beverages while increasing the intake of plant-based foods. The diet’s high fiber content promotes satiety, regulates energy intake, and supports metabolic health, addressing key drivers of obesity [84,85,86,87]. This review offers a comprehensive evaluation of the PHD, integrating global nutritional guidelines and assessing its applicability to pediatric populations. A key strength lies in its multidimensional approach, which considers health, environmental, and cultural factors. Additionally, the detailed analysis of nutrient adequacy, compared with international organization references, and proposed adaptations provide actionable insights for implementing the PHD in diverse contexts. Nonetheless, this review’s reliance on secondary data and theoretical modeling limits its empirical validation. The lack of longitudinal studies on the PHD’s impact on children’s growth and development represents a critical research gap. Furthermore, the variability in adherence rates across different populations highlights the need for standardized metrics and robust evaluation frameworks to assess the diet’s effectiveness comprehensively.

## 5. Conclusions

The Planetary Health Diet (PHD) is a model proposed by the EAT-Lancet Commission to promote human health and protect the environment. It aims to address global challenges such as climate change, biodiversity loss, environmental degradation, and the increasing prevalence of non-communicable diseases. A key question that arises is whether the principles of this diet can be applied to children and adolescents. In this context, adapting the PHD to pediatric populations would offer a good opportunity to address the dual challenges of childhood obesity and environmental sustainability. This review emphasizes the need to tailor the PHD to meet children’s unique nutritional requirements, including ensuring an adequate energy intake, optimizing the nutrient bioavailability through strategic food pairings, and incorporating fortified foods or supplements for critical vitamins and minerals, such as iron, zinc, and vitamin D. Such adaptations must also respect cultural and regional contexts to ensure relevance and acceptance.

The core principles of the PHD revolve around several key dietary changes. One of the main aspects is reducing the consumption of animal products. This reduction is not only due to the environmental impact of meat production but also because of the health risks associated with excessive intake of red and processed meat, which are high in saturated fats. Another key point is increasing the intake of vegetables, fruits, nuts, and seeds. These foods are nutrient-dense and environmentally sustainable. They are rich in essential vitamins, minerals, and fiber, making them supportive of children’s health. The PHD also recommends incorporating wholegrain cereals, since they provide a good source of energy, fiber, and vitamin B. Ensuring diversity and increasing the share of wholegrain products helps children have a balanced and nutritious diet. Lastly, the PHD advocates for limiting the consumption of sugar and highly processed foods, which are often high in unhealthy fats, sugar, and salt. The excessive intake of such foods has been linked to the rise in obesity and type 2 diabetes in children.

To achieve these goals, family-level interventions and school meal programs play a pivotal role. Families can be supported through educational initiatives that promote balanced meal planning, the efficient use of plant-based protein sources, and strategies to introduce sustainable dietary practices early in life. Simultaneously, school meal programs offer a structured opportunity to align with the PHD principles by incorporating nutrient-dense, locally sourced ingredients, minimizing food waste, and offering meals that are both nutritionally adequate and appealing to children. Integrating nutrition education into school curricula can further reinforce these principles, fostering lifelong habits that balance health and sustainability. Collaboration among policymakers, healthcare professionals, educators, and families is essential for the successful implementation of a pediatrically adapted PHD. Regional adaptations leveraging local food systems can enhance its feasibility, accessibility, and cultural resonance, making sustainable diets a practical reality.

Future research should prioritize longitudinal studies to evaluate the health and environmental impacts of pediatric PHD adaptations while addressing potential barriers, such as cost, food accessibility, and acceptance. With careful planning, gradual adjustments, and educational efforts through the integration of nutritional health and environmental stewardship, the PHD can become a transformative model not only for combating childhood obesity but also for fostering a sustainable and equitable future for the next generation.

## Figures and Tables

**Table 1:** Macro and micro recommended nutrients intake for adults in physiological condition and the Planetary Health Diet model.

Authorities’ Recommendations	Energy (Kcal/die)		Water (mL/die)		Carbs (%En)	Sugar (g/die)	Fiber (g/die)	Total Lipids (%En)	Sat. Fatty Acids (%En)	PUFA (%En)	LC-PUFA n-3	Chol.	Prot. (g/kg/die)	Vit B6 (mg/die)		Vit B9 (µg/die)	Vit B12 (µg/die)	Vit C (mg/die)		Vit A (µg/die)		Vit D (µg/die)	Vit E (mg/die)		Sodio (g/die)	Pot. (mg/die)	Magnesium (mg/die)		Calcium (mg/die)	Phosp (mg/die)	Iron (mg/die)		Zinc (mg/die)		Iodine (µg/die)	Selenium (µg/die)	
	M	F	M	F	M/F	M/F	M/F	M/F	M/F	M/F	M/F	M/F	M/F	M	F	M/F		M	F	M	F	M/F	M	F	M/F	M/F	M	F	M/F	M/F	M	F	M	F	M/F	M	F
National level—LARN 2024 [10]	2330 (AR) [10]	1881 (AR) [10]	2500 (AI) [10]	2000 (AI) [10]	Prefer starchy food sources with a low glycemic index, especially when carbohydrate intake approaches the upper limit of RI. However, limit foods where glycemic index reduction is achieved by increasing fructose or fat content (SDT); 45–60 (RI) [10]	Limit sugar intake to <15% En. A total intake >25% En is considered potentially linked to adverse health events. Limit the use of fructose as a sweetener or drinks containing it, as well as corn syrup (SDT) [10]	Prefer foods naturally rich in dietary fiber as a source of fiber. In adults, consume at least 25 g/day of dietary fiber even in case of energy intake <2000 kcal/day (SDT); 12.6–16.7 g/1000 Kcal (AI) [10]	20–35 (RI) [10]	<10 (SDT) [10]	5–10 (RI) [10]	EPA-DHA 250 mg/die (AI); 0.5–2 %En (RI) [10]	/	0,71 (AR); 0.90 (PRI) [10]	1.4 (AR); 1.6 (PRI); 12 (UL) [10]	1.2 (AR); 1.4 (PRI); 12 (UL) [10]	250 (AR); 330 (PRI); 1000 (UL) [10]	4 (AI) [10]	75 (AR); 105 (PRI) [10]	60 (AR); 85 (PRI) [10]	570 (AR); 750 (PRI); 3000 (UL) [10]	490 (AR); 650 (PRI); 3000 (UL) [10]	10 (AR); 15 (PRI); 100 (UL) [10]	13 (AI), 300 (UL) [10]	12 (AI); 300 (UL) [10]	1.5 (AI); 2 (SDT) [10]	4500 (SDT) [10]	350 (AI) [10]		750 (AR); 950 (PRI); 2500 (UL) [10]	550 (AI) [10]	7 (AR); 10 (PRI) [10]	10 (AR); 18 (PRI) [10]	10 (AR); 12 (PRI) [10]	8 (AR); 9 (PRI) [10]	150 (AI); 600 (UL) [10]	55 (AI); 200 (UL) [10]	
European level—EFSA [11,12,13,14,15,16,17,18,19,20,21,22,23,24,25,26,27,28,29,30,31]	2390 [11]	2390 [11]	2500 (AI) [31]	2000 (AI) [31]	45–60 (AMDR) [13]	/	25 (AI) [13]	20–35 (RI) [12]	as least as possible [12]	/	/	/	0.83 (AR) [14]	1.5 (AR); 1.7 (PRI); 12 (UL) [15]	1.3 (AR); 1.6 (PRI); 12 (UL) [15]	250 (folate equivalents) (AR); 330 (folate equivalents) (PRI); 1000 (UL) [16]	4 (AI) [17]	90 (AR); 110 (PRI) [18]	80 (AR); 95 (PRI) [18]	570 (AR); 750 (PRI); 3000 (UL) [19]	490 (AR); 650 (PRI); 3000 (UL) [19]	15 (AI); 100 (UL) [21]	13 (AI); 300 (UL) [20]	11 (AI); 300 (UL) [20]	2 (AI) [22]	3500 (AI) [23]	350 (AI) [24]	300 (AI) [24]	860 (AR) (18–24 yo); 750 (AR) (>25 yo); 1000 (PRI) (18–24 yo); 950 (PRI) (>25 yo) [25]	524 (PRI); 550 (AI) [26]	6 (AR); 11 (PRI) [27]	7 (AR); 16 (PRI) [27]	7.5 (AR), 9.4 (PRI), level of phytate intake 300 mg/die; 25 (UL) [28]	6.2 (AR), 7.5 (PRI) level of phytate intake 300 mg/die; 25 (UL) [28]	150 (AI) [29]	70 (AI); 225 (UL) [30]	
International level—WHO and WHO/FAO [32,33,34,35,36,37,38,39,40]	2500 [32]	2000 [32]	3700 (AI) [40]	2700 (AI) [40]	55–75 (AMDR) [38]	<10% En (UL) [37]	>25 (AI) [38]	20-35 (RI) [34,35,36]	<10 (AI) [34,35,36]	6–11 (RI) [34,35,36]	/	<300 (AI) [34,35,36]	0.83 (AR) [33]	1.3 (PRI) [39]		320 (folate equivalents) (AR); 400 (folate equivalents) (PRI); 1000 (UL) [39]	2 (AR); 2.4 (PRI) [39]	30 (AR); 45 (PRI) [39]		300 (AR); 600 (PRI) [39]	270–300 (AR); 500–600 (PRI) [39]	5 (PRI) (18–50 yo); 10 (PRI) (51–65 yo) [39]	10 (AI) [39]	7.5 (AI) [39]	2 h (SDT) [39]	3510 (PRI) [39]	260 (PRI); 350 (UL) [39]	220 (PRI); 350 (UL) [39]	1000 (AI); 3000 (UL) [39]	/	9.1 (PRI) (iron bioavailability 15%); 27.4 (PRI) (iron bioavailability 5%) [39]	19.6 (PRI) (iron bioavailability 15%); 58.8 (PRI) (iron bioavailability 5%) [39]	4.2 (PRI) high availability; 45 (UL) [39]	3 (PRI) high availability; 45 (UL) [39]	150 (AI) [39]	34 (PRI); 400 (UL) [39]	26 (PRI); 400 (UL) [39]
Planetary health diet [5]	2490				43.3	13.5	34.7	40.03	10.7	32.77	0.93	177	100.44	2.5		449.37	4.55	149		1025 (ret eq)		1.52	35.5		0.77	3289.3	443.6		1004.3	1847	15.5		12.4		61.4	36.11	

In order to compare whether the PHD model meets the needs of an adult subject (in terms of energy, macro- and micronutrients), the following methodology was adopted: referring to national and international organizations/guidelines, the nutrient requirements for the adult population were identified from the latest version of the ‘Reference Intake Levels for Nutrients and Energy’ (LARN) 2024 [10] (national level), from the EFSA (European level) [11,12,13,14,15,16,17,18,19,20,21,22,23,24,25,26,27,28,29,30] and finally from FAO and WHO (international level) [31,32,33,34,35,36,37,38]. It should be specified that the reference values taken into consideration were the nutrient requirements for an adult of average height and weight, in physiological condition. The methodology adopted is described in the section: materials and methods. To define the energy requirement (Kcal/die), the relative reference intake levels of a male subject aged between 30 and 59 years, with an average height of 1.75 m and a PAL (physical activity level) of 1.4 are considered. The average values considered for female subjects are 1.65 m, aged between 30 and 59 years and PAL 1.4. (Adequate Intake = AI; Acceptable macronutrient distribution range = AMDR; Suggested Dietary Intake = SDT; Reference intake range for macronutrients = RI; Average requirement = AR; Population reference intake = PRI; Tolerable upper intake level = UL; Male = M; Female = F; Carbohydrates = Carbs; Saturated Fatty Acid = Sat. fatty acids; Potassium = Pot; Phosphorus = Phosp; years old = yo.).

## Supplementary Materials

The following supporting information can be downloaded at https://www.mdpi.com/article/10.3390/nu16244316/s1: Table S1: Average energy requirement (AR) for Italian males and females in the age range of 2–17 years; Table S2: Reference lipid intake levels for Italian children and adolescents, aged 2–3 and 4–17 years; Table S3: Reference carbohydrate intake levels for Italian children and adolescents, aged 2–3 and 4–17 years; Table S4: Population reference intake (PRI) of protein for Italian males and females, aged 2–17 years; Table S5: Daily reference intake for the population (PRI) or adequate intake (AI) of selected vitamins for Italian males and females, aged 2–17 years; Table S6: Daily reference intake for the population (PRI), adequate intake (AI), or nutritional objective for prevention (SDT) of selected minerals for Italian males and females, aged 2–17 years; Table S7: Average energy requirement (AR) for European males and females, aged 2–17 years, based on different activity levels (1.2–1.4–1.6–1.8–2.0); Table S8: Dietary reference values (DRV) for lipids for European children and adolescents, aged 2–3 and 4–17 years; Table S9: Reference intake levels of carbohydrates for European children and adolescents, aged 2–3 and 4–17 years; Table S10: Population reference intake (PRI) of proteins for European males and females, aged 2–17 years, based on different body weights; Table S11: Daily reference intake for the population (PRI) or adequate intake (AI) of selected vitamins for European males and females, aged 2–17 years; Table S12: Daily reference intake for the population (PRI), adequate intake (AI), or nutritional objective for prevention (SDT) of selected minerals for European males and females, aged 2–17 years; Table S13: Global energy requirement for males and females, aged 2–17 years, based on different activity levels (light, moderate, and heavy); Table S14: Reference lipid intake levels for children and adolescents, aged 2–3 and 4–17 years; Table S15: Reference carbohydrate intake levels for Italian children and adolescents, aged 2–3 and 4–17; Table S16: Reference protein intake levels for males and females, aged 2–17 years, based on different body weights; Table S17: Recommended nutrient intake (RNI) of selected vitamins for males and females, aged 2–17 years; Table S18: Recommended nutrient intake of selected minerals for males and females, aged 2–17; Table S19: Comparison with reference intake levels for the Italian population (LARN, 2024) for males, aged 2–17 years; Table S20: Comparison with reference intake levels for the Italian population (LARN, 2024) for females, aged 2–17 years; Table S21: Comparison with reference intake levels for the European population (EFSA) for males, aged 2–17 years; Table S22: Comparison with reference intake levels for the European population (EFSA) for females, aged 2–17 years; Table S23: Comparison with reference intake levels for the global population (WHO/FAO) for males, aged 2–17 years; Table S24: Comparison with reference intake levels for the global population (WHO/FAO) for females, aged 2–17 years; Table S25: Adequacy of PHD in meeting reference intake levels according to LARN, EFSA, and WHO/FAO recommendations for females and males, aged 2–17 years; Table S25a. Adequacy of PHD in meeting energy recommendation of females and males, 2 to 17 years, according to LARN, EFSA, and FAO/WHO. Table S25b. Adequacy of PHD in meeting dietary fiber recommendation of females and males, 2 to 17 years, according to LARN, EFSA and FAO/WHO. Table S25c. Adequacy of PHD in meeting protein recommendation of females and males, 2 to 17 years, according to LARN, EFSA and FAO/WHO. Table S25d. Adequacy of PHD in meeting vitamin B6 recommendation of females and males, 2 to 17 years, according to LARN, EFSA and FAO/WHO. Table S25e. Adequacy of PHD in meeting folate recommendation of females and males, 2 to 17 years, according to LARN, EFSA and FAO/WHO. Table S25f. Adequacy of PHD in meeting Vitamin B12 recommendation of females and males, 2 to 17 years, according to LARN, EFSA and FAO/WHO. Table S25g. Adequacy of PHD in meeting Vitamin C recommendation of females and males, 2 to 17 years, according to LARN, EFSA and FAO/WHO. Table S25h. Adequacy of PHD in meeting Vitamin A recommendation of females and males, 2 to 17 years, according to LARN, EFSA and FAO/WHO. Table S25i. Adequacy of PHD in meeting Vitamin E recommendation of females and males, 2 to 17 years, according to LARN, EFSA and FAO/WHO. Table S25l. Adequacy of PHD in meeting Vitamin D recommendation of females and males, 2 to 17 years, according to LARN, EFSA and FAO/WHO. Table S25m. Adequacy of PHD in meeting Sodium recommendation of females and males, 2 to 17 years, according to LARN and EFSA. Table S25n. Adequacy of PHD in meeting Potassium recommendation of females and males, 2 to 17 years, according to LARN and EFSA. Table S25o. Adequacy of PHD in meeting Magnesium recommendation of females and males, 2 to 17 years, according to LARN, EFSA and FAO/WHO. Table S25p. Adequacy of PHD in meeting Calcium recommendation of females and males, 2 to 17 years, according to LARN, EFSA and FAO/WHO. Table S25q. Adequacy of PHD in meeting Phosphorus recommendation of females and males, 2 to 17 years, according to LARN and EFSA. Table S25r. Adequacy of PHD in meeting Iron recommendation of females and males, 2 to 17 years, according to LARN, EFSA and FAO/WHO. Table S25s. Adequacy of PHD in meeting Zinc recommendation of females and males, 2 to 17 years, according to LARN, EFSA and FAO/WHO. Table S25t. Adequacy of PHD in meeting Iodine recommendation of females and males, 2 to 17 years, according to LARN, EFSA and FAO/WHO. Table S25u. Adequacy of PHD in meeting Selenium recommendation of females and males, 2 to 17 years, according to LARN, EFSA and FAO/WHO.
