# Supplementary material for: Planetary Health Diet for Childhood Obesity Prevention: Integrating Nutritional Health with Environmental Stewardship
Source: Nutrients. 2024 Dec 13;16(24):4316. doi: 10.3390/nu16244316 (PMC11676438; doi:10.3390/nu16244316)
Supplement: Supplementary file 1 [file nutrients-16-04316-s001.zip › nutrients-3367881-supplementary.pdf]

Supplementary Materials

Reference Intake Levels for the Italian Population [10].

Table S1. Average energy requirement (AR) for Italian males and females in the age range 2-17 years, based on different activity levels (1,2 – 1,4 – 1,6 – 1,8 – 2,0), proposed by LARN 2024.

AGE (years)	MALE				
	Energy requirements (kcal/day) for a PAL of:				
	1,2	1,4	1,6	1,8	2,0
2-3	860	1075	1230	1380	
3-4	960	1200	1380	1550	
4-5	1010	1270	1450	1630	
5-6	1060	1340	1530	1720	
6-7	1110	1410	1610	1810	
7-8	1170	1490	1700	1910	
8-9	1240	1570	1790	2020	2240
9-10	1330	1660	1900	2140	2380
10-11		1750	2000	2250	2500
11-12		1850	2110	2380	2640
12-13		1970	2250	2530	2820
13-14		2120	2420	2720	3020
14-15		2270	2600	2920	3250
15-16		2400	2740	3090	3430
16-17		2510	2870	3230	3580
17-18		2580	2950	3320	3690
AGE (years)	FEMALE				
	Energy requirements (kcal/day) for a PAL of:				
	1,2	1,4	1,6	1,8	2,0
2-3	920	1000	1150	1290	
3-4	1030	1120	1280	1440	
4-5	1090	1180	1350	1520	
5-6	1140	1240	1410	1590	
6-7	1210	1300	1480	1670	
7-8	1270	1360	1560	1750	
8-9	1350	1450	1650	1860	2070
9-10	1430	1550	1770	1990	2210
10-11		1620	1850	2090	2320
11-12		1710	1960	2200	2440
12-13		1800	2060	2320	2580
13-14		1890	2150	2420	2690
14-15		1950	2230	2510	2790
15-16		2000	2280	2570	2860
16-17		2030	2310	2600	2890
17-18		2040	2340	2630	2920

Legend Table 1a: PAL, physical activity level. Average energy requirements rounded to 10 kcal/day. All values reference to the end of the sixth month (midpoint) of the year under consideration.

Table S2. Lipids reference intake levels for Italian children and adolescents, aged 2-3 and 4-17 years, proposed by LARN 2024.

AGE (years)	Total fats (% En)	SFA (% En)	AG trans (% En)	MUFA (% En)	PUFA (% En)	PUFA n-6 (% En)	PUFA n-3 (% En)
2-3	35-40	< 10	As low as possible	By differenc e	5-10	4-8	0,5-2 EPA-DHA 250 mg/day 2 yeas: + DHA 100 mg/day
4-17	20-35	< 10	As low as possible	By differenc e	5-10	4-8	0,5-2 EPA-DHA 250 mg/day

Legend Table 2a. %En: percentage of total dietary energy; SFA: saturated fatty acids; PUFA: polyunsaturated fatty acids; PUFA n-6: n-6 series polyunsaturated fatty acids; PUFA n-3: n-3 series polyunsaturated fatty acids; EPA: eicosapentaenoic acid; DHA: docosahexaenoic acid. The amount of monounsaturated fatty acids (MUFAs) to be consumed in diets is calculated by difference, considering SDT for SFAs and RI for PUFAs.

Table S3. Carbohydrates reference intake levels for Italian children and adolescents, aged 2-3 and 4-17 years, proposed by LARN 2024

AGE (years)	Total carbohydrates (% En)	Sugar (% En)	Dietary fiber (g/day)
2-17	45-60	< 15	8,4 g/1000 kcal

Legend Table 3a. %En: percentage of total dietary energy; sugar: include simple sugars naturally found in milk, fruits and vegetables and added sugars.

Table S4. Proteins population reference intake (PRI) for Italian males and females, aged 2-17 years, based on different body weight, proposed by LARN 2024.

AGE (years)	MALE	
	g/kg/day	g/day
2-3	1,05	14,0
3-4	0,97	14,8
4-5	0,93	16,1
5-6	0,92	17,8
6-7	0,96	20,8
7-8	0,98	23,6
8-9	0,99	26,4
9-10	0,99	29,3
10-11	0,98	32,2
11-12	0,98	36,0
12-13	0,97	40,4
13-14	0,97	46,0
14-15	0,96	51,6
15-16	0,95	55,9
16-17	0,94	59,3
17-18	0,93	61,5
AGE (years)	FEMALE	
	g/kg/day	g/day
2-3	1,05	13,3
3-4	0,97	14,6
4-5	0,93	16,0
5-6	0,92	17,6
6-7	0,96	20,4
7-8	0,98	23,1
8-9	0,99	26,2
9-10	0,99	29,7
10-11	0,98	33,3
11-12	0,97	37,5
12-13	0,96	41,9
13-14	0,95	45,5
14-15	0,94	48,4
15-16	0,92	49,6
16-17	0,91	50,3
17-18	0,90	50,7

Legend Table 4a. All values reference to the end of the sixth month (midpoint) of the year under consideration.

Table S5a. Daily reference intake for the population (**PRI in bold**) or adequate intake (*AI in italics*) of some selected vitamins for Italian male and female, aged 2-17 years, proposed by LARN 2024.

AGE (years)	Vit.B6 (mg)	Folate (µg)	Vit.B12 (µg)	Vit.C (mg)	Vit.A (µg)	Vit.E (mg)	Vit.D (µg)
2-3	**0,6**	**120**	*1,4*	**35**	**250**	5	**15**
4-6	**0,7**	**140**	*1,7*	**45**	**300**	6	**15**
7-10	**1,0**	**200**	*2,5*	**60**	**400**	8	**15**
AGE (years)	MALE						
11-14	**1,3**	**270**	*3,4*	**90**	**600**	*11*	**15**
15-17	**1,6**	**330**	*4,0*	**105**	**750**	*13*	**15**
AGE (years)	FEMALE						
11-14	**1,3**	**270**	*3,3*	**80**	**600**	*11*	**15**
15-17	**1,4**	**330**	*4,0*	**85**	**650**	*12*	**15**

Legend Table 5a. Vitamin A is expressed in µg of retinol equivalents (1 RE = 1 µg of retinol = 6 µg of beta-carotene = 12 µg of other provitamin carotenoids). Vitamin D is expressed as cholecalciferol (1 µg of cholecalciferol = 40 IU of vitamin D); the PRI considers both food intake and the endogenous synthesis of the skin. Vitamin E is expressed in alpha-tocopherol equivalents (1 alpha-TE = 1 mg RRR-tocopherol = 1.5 IU = 2 mg beta-tocopherol = 3 mg gamma-tocotrienol = 10 mg gamma-tocopherol).

Table S6a. Daily reference intake for the population (PRI in bold), adequate intake (*AI in italics*) or nutritional objective for prevention (<u>SDT underlined</u>) of some selected minerals for Italian male and female, aged 2-17 years, proposed by LARN 2024.

AGE (years)	Na (g)	K (mg)	Mg (mg)	Ca (mg)	P (mg)	Fe (mg)	Zn (mg)	I (µg)	Se (µg)
2-3	*0,5*	<u>1500</u>	*120*	**510**	295	**8**	**5**	*100*	*20*
4-6	*0,6*	<u>1900</u>	*150*	**900**	520	**11**	**6**	*100*	*25*
7-10	*1,0*	<u>2700</u>	*220*	**1040**	600	**13**	**8**	*100*	*35*
AGE (years)	MALE								
11-14	*1,5*	<u>4500</u>	*290*	**1150**	*660*	**10**	**12**	*130*	*50*
15-17	*1,5*	<u>4500</u>	*380*	**1150**	*660*	**13**	**12**	*130*	*55*
AGE (years)	FEMALE								
11-14	*1,5*	<u>4500</u>	*290*	**1150**	*660*	**10/18**	**9**	*130*	*50*
15-17	*1,5*	<u>4500</u>	*310*	**1150**	*660*	**18**	**9**	*130*	*55*

Legend Table 6a. For iron, in the 11-14 year age group, the second PRI values refer to adolescents who menstruate.

Reference Intake Levels for the European Population (EFSA) [11-30]

Table S7. Average energy requirement (AR) for European males and females, aged 2-17 years, based on different activity levels (1,2 – 1,4 – 1,6 – 1,8 – 2,0), proposed by EFSA 2013.

AGE (years)	MALE			
	Energy requirements (kcal/day) for a PAL of:			
	1,4	1,6	1,8	2,0
2	1027			
3	1170			
4	1266	1433	1624	
5	1338	1529	1720	
6	1409	1600	1815	
7	1505	1720	1935	
8	1600	1815	2054	
9	1672	1935	2174	
10		1935	2174	2412
11		2030	2293	2556
12		2174	2436	2723
13		2341	2627	2914
14		2508	2818	3129
15		2699	3033	3368
16		2842	3201	3559
17		2938	3296	3678
AGE (years)	FEMALE			
	Energy requirements (kcal/day) for a PAL of:			
	1,4	1,6	1,8	2,0
2	955			
3	1099			
4	1170	1338	1505	
5	1242	1409	1600	
6	1314	1505	1696	
7	1385	1600	1791	
8	1481	1696	1887	
9	1576	1791	2006	
10		1815	2054	2269
11		1911	2150	2388
12		2006	2245	2508
13		2102	2365	2627
14		2174	2436	2723
15		2221	2508	2795
16		2269	2532	2818
17		2269	2556	2842

Legend Table S7: PAL, physical activity level. EFSA reports energy in MJ/day, for clarity the authors converted values to kcal/day.

Table S8. Lipids dietary reference values (DRV) for European children and adolescents, aged 2-3 and 4-17 years, proposed by EFSA 2010.

AGE (years)	Total fats (% En)	SFA (% En)	AG trans (% En)	MUFA (% En)	PUFA (% En)	PUFA n-6 (% En)	PUFA n-3 (% En)
2-3	35-40	As low as possible	As low as possible	No DRV	No DRV	No DRV	No DRV
4-17	20-35	As low as possible	As low as possible	No DRV	No DRV	No DRV	No DRV

Legend Table S8. %En: percentage of total dietary energy; SFA: saturated fatty acids; PUFA: polyunsaturated fatty acids; PUFA n-6: n-6 series polyunsaturated fatty acids; PUFA n-3: n-3 series polyunsaturated fatty acids.

Table S9. Carbohydrates reference intake levels for European children and adolescents, aged 2-3 and 4-17 years, proposed by EFSA 2010.

AGE (years)	Total carbohydrates[a] (% En)	Dietary fiber[b] (g/day)
2-3	45-60	10
4-6	45-60	14
7-10	45-60	16
11-14	45-60	19
15-17	45-60	21

Legend Table S9. % En: percentage of total dietary energy.
(a)reference intake range;
(b)adequate intake.

Table S10. Proteins population reference intake (PRI) for European males and females, aged 2-17 years, based on different body weight, proposed by EFSA 2012.

AGE (years)	MALE	
	**g/kg/day**	**g/day**
2-3	0,97	12
3-4	0,90	13
4-5	0,86	15
5-6	0,85	16
6-7	0,89	19
7-8	0,91	22
8-9	0,92	25
9-10	0,92	28
10-11	0,91	31
11-12	0,91	34
12-13	0,90	37
13-14	0,90	42
14-15	0,89	47
15-16	0,88	52
16-17	0,87	56
17-18	0,86	58
AGE (years)	FEMALE	
	**g/kg/day**	**g/day**
2-3	0,97	12
3-4	0,90	13
4-5	0,86	14
5-6	0,85	16
6-7	0,89	19
7-8	0,91	22
8-9	0,92	25
9-10	0,92	28
10-11	0,91	31
11-12	0,90	34
12-13	0,89	38
13-14	0,88	42
14-15	0,87	45
15-16	0,85	46
16-17	0,84	47
17-18	0,83	48

Table S11. Daily reference intake for the population (**PRI in bold**) or adequate intake (*AI in italics*) of some selected vitamins for European male and female, aged 2-17 years, proposed by EFSA.

AGE (years)	Vit.B6 (mg)	Folate (µg)	Vit.B12 (µg)	Vit.C (mg)	Vit.A (µg)	Vit.E (mg)	Vit.D (µg)
2-3	**0,6**	**120**	*1,5*	**20**	**250**	*6*	*15*
4-6	**0,7**	**140**	*1,5*	**30**	**300**	*9*	*15*
7-10	**1,0**	**200**	*2,5*	**45**	**400**	*9*	*15*
AGE (years)	MALE						
11-14	**1,4**	**270**	*3,5*	**70**	**600**	*13*	*15*
15-17	**1,8**	**330**	*4,0*	**100**	**750**	*13*	*15*
AGE (years)	FEMALE						
11-14	**1,4**	**270**	*3,5*	**70**	**600**	*11*	*15*
15-17	**1,6**	**330**	*4,0*	**90**	**650**	*11*	*15*

Legend Table S11. Vitamin A is expressed in µg of retinol equivalents (1 RE = 1 µg of retinol = 6 µg of beta-carotene = 12 µg of other provitamin carotenoids). Vitamin E is expressed in alpha-tocopherol equivalents (1 alpha-TE = 1 mg RRR-tocopherol = 1.5 IU = 2 mg beta-tocopherol = 3 mg gamma-tocotrienol = 10 mg gamma-tocopherol); Age ranges for alpha-tocopherol requirements: < 3 years; from 3 to < 10 years; ≥ 10 years. Vitamin D: under conditions of assumed minimal cutaneous vitamin D synthesis. In the presence of endogenous cutaneous vitamin D synthesis, the requirement for dietary vitamin D is lower or may be even zero.

Table S12. Daily reference intake for the population (**PRI in bold**), adequate intake (*AI in italics*) or nutritional objective for prevention (SDT underlined) of some selected minerals for European male and female, aged 2-17 years, proposed by EFSA.

AGE (years)	Na (g)	K (mg)	Mg (mg)	Ca (mg)	P (mg)	Fe (mg)	Zn (mg)	I (µg)	Se (µg)
2-3	*1,1*	*800*	*170 (2 y)*<br>*230 (3 y)*	**388**	*250*	**7**	*3,6*	*90*	*15*
4-6	*1,3*	*1100*	*230*	**681**	*440*	**7**	*4,6*	*90*	*20*
7-10	*1,7*	*1800*	*230 (7-9)*<br>*300 (10)*	**672**	*440*	**11**	*6,2*	*90*	*35*
AGE (years)	MALE								
11-14	*2,0*	*2700*	*300*	**944**	*640*	**11**	*8,8*	*120*	*55*
15-17	*2,0*	*3500*	*300*	**965**	*640*	**11**	*11,8*	*130*	*70*
AGE (years)	FEMALE								
11-14	*2,0*	*2700*	*250*	**944**	*640*	**11 (11 y)**<br>**13 (12-14 y)**	*8,9*	*120*	*55*
15-17	*2,0*	*3500*	*250*	**965**	*640*	**13**	*9,9*	*130*	*70*

Legend Table S12. Calcium dietary requirement are calculated as: [(urinary losses + faecal losses + dermal losses) + calcium accretion in bone] / fractional absorption. Average requirements for zinc is estimated from the physiological requirement and assuming an absorption efficiency of 30 % from a mixed diet; values were rounded to the nearest 0.1.

Reference Intake Levels for the Global Population (WHO/FAO) [31-38]

Table S13. Energy requirement for worldwide males and females, aged 2-17 years, based on different activity levels (light, moderate and heavy) proposed by FAO/WHO/UNU 2001.

AGE (years)	MALE		
	Energy requirements (kcal/day) for a PAL of:		
	Light physical activity	Moderate physical activity	Heavy physical activity
2-3		1125	
3-4		1250	
4-5		1350	
5-6		1475	
6-7	1350	1575	1800
7-8	1450	1700	1950
8-9	1550	1825	2100
9-10	1675	1975	2275
10-11	1825	2150	2475
11-12	2000	2350	2700
12-13	2175	2550	2925
13-14	2350	2775	3175
14-15	2550	3000	3450
15-16	2700	3175	3650
16-17	2825	3325	3825
17-18	2900	3400	3925
AGE (years)	FEMALE		
	Energy requirements (kcal/day) for a PAL of:		
	Light physical activity	Moderate physical activity	Heavy physical activity
2-3		1050	
3-4		1150	
4-5		1250	
5-6		1325	
6-7	1225	1425	1650
7-8	1325	1550	1775
8-9	1450	1700	1950
9-10	1575	1850	2125
10-11	1700	2000	2300
11-12	1825	2150	2475
12-13	1925	2275	2625
13-14	2025	2375	2725
14-15	2075	2450	2825
15-16	2125	2500	2875
16-17	2125	2500	2875
17-18	2125	2500	2875

Legend Table S13. PAL, physical activity level.

Table S14. Lipids reference intake levels for children and adolescents, aged 2-3 and 4-17 years, proposed by WHO, 2023.

	Total fats (% En)	SFA (% En)	AG trans (% En)
WHO, 2023	Not reported*	< 10	< 1

Legend Table S14. WHO state that the evidence was considered insufficient to support the formulation of a recommendation for totsl fats intake for children owing to the limited number of studies and inconsistent results identified for children, and the conclusion that the adult data could not reasonably be extrapolated to children given the unique energy requirements for optimal growth and development throughout childhood and adolescence.

Table S15. Carbohydrates reference intake levels for Italian children and adolescents, aged 2-3 and 4-17, proposed by WHO.

	Total Carbohydrates (% En)	Sugar (% En)	Dietary fibre (g/d)
WHO, 2003	55-75		
WHO, 2015		Free sugar < 10	
WHO, 2023			2–5 years old, at least 15 g per day<br><br>6–9 years old, at least 21 g per day<br><br>10 years or older, at least 25 g per day.

Table S16. Proteins reference intake levels for males and females, aged 2-17 years, based on different body weight, proposed by WHO/FAO 2007.

AGE (years)	MALE	
	**g/kg/day**	**g/day**
2-3	0,97	12,9
3-4	0,90	13,8
4-5	0,86	14,9
5-6	0,85	16,5
6-7	0,89	19,3
7-8	0,91	21,9
8-9	0,92	24,6
9-10	0,92	27,2
10-11	0,91	29,9
11-12	0,90	33,0
12-13	0,90	37,4
13-14	0,90	42,7
14-15	0,90	48,3
15-16	0,87	51,2
16-17	0,87	54,9
17-18	0,87	57,5
AGE (years)	FEMALE	
	**g/kg/day**	**g/day**
2-3	0,97	12,3
3-4	0,90	13,5
4-5	0,86	14,8
5-6	0,85	16,2
6-7	0,89	18,9
7-8	0,91	21,5
8-9	0,92	24,4
9-10	0,92	27,6
10-11	0,91	30,9
11-12	0,89	34,4
12-13	0,89	38,8
13-14	0,89	42,6
14-15	0,89	45,8
15-16	0,84	45,3
16-17	0,84	46,5
17-18	0,84	47,3

Table S17. Recommended nutrient intake (RNI) of some selected vitamins for male and female, aged 2-17 years, proposed by WHO 2004.

AGE (years)	Vit.B6 (mg)	Folate (mg)	Vit.B12 (µg)	Vit.C (mg)	Vit.A (µg)	Vit.E (mg)	Vit.D (µg)
2-3	0,5	160	0,9	30[a]	400	5[b]	5
4-6	0,6	200	1,2	30[a]	450	5[b]	5
7-9	1,0	300	1,8	30[a]	500	7[b]	5
AGE (years)	MALE						
10-18	1,3	400	2,4	30[a]	600	10	5
AGE (years)	FEMALE						
10-18	1,2	400	2,4	30[a]	600	7.5	5

Legend Table S17. Vitamin A is expressed in µg of retinol equivalents (1 RE = 1 µg of retinol = 6 µg of beta-carotene = 12 µg of other provitamin carotenoids). Vitamin E is expressed in alpha-tocopherol equivalents (1 alpha-TE = 1 mg RRR-tocopherol = 1.5 IU = 2 mg beta-tocopherol = 3 mg gamma-tocotrienol = 10 mg gamma-tocopherol); data were considered insufficient to formulate recommendations for this vitamin so that "acceptable intakes" are listed instead. This represents the best estimate of requirements, based on the currently acceptable intakes that support the known function of this vitamin.

a) Arbitrary values
b) Values based on a proportion of the adult acceptable intakes.

Table S18. Recommended nutrient intake of some selected minerals for male and female, aged 2-17, proposed by WHO 2004.

AGE (years)	Na (g)	K (mg)	Mg (mg)	Ca (mg)	P (mg)	Fe (mg)	Zn (mg)	I (µg)	Se (µg)
2-3	nr	nr	60	500	nr	5	4,1	75	17
4-6	nr	nr	73	600	nr	5	5,1	110	21
7-9	nr	nr	100	700	nr	7	5,6	100	21
AGE (years)	MALE								
10-18	nr	nr	250	1300[a]	nr	12 (10-14 y)<br>16 (15-18 y)	9,7	135 (10-11 y)<br>110 (12+ y)	34
AGE (years)	FEMALE								
10-18	nr	nr	230	1300[a]	nr	12 (10-14 y)[b]<br>28 (10-14 y)<br>26 (15-18 y)	7,8	140 (10-11 y)<br>100 (12+ y)	26

Legend Table S18. For the iron reccomended nutrient intake was considered the 12% bioavailability. For the zinc reccomended nutrient intake was considered the moderate bioavailability. Nr: not reported.

a) Particularly during the growth spurt.
b) Non-menstruating adolescents.

Table S19: Comparison with Reference Intake Levels for the Italian Population (LARN, 2024) for Males, aged 2-17 years.

YEARS (AGE)	2-3	3-4	4-5	5-6	6-7	7-8	8-9	9-10	10-11	11-12	12-13	13-14	14-15	15-16	16-17	17-18
PHD intake	2500 kcal/day															
Energy requirements	1230	1380	1450	1530	1610	1700	1790	1900	2000	2110	2250	2420	2600	2740	2870	2950

Dietary fiber requirements (g/day)	10	12	12	13	14	14	15	16	17	18	19	20	22	23	24	25
PHD intake	35 g/day															
Intake adjusted for Energy requirements	17	19	20	21	23	24	25	27	28	30	32	34	36	38	40	41

Proteins requirements (g/day)	14	14.8	16.1	17.8	20.8	23.6	26.4	29.3	32.2	36	40.6	46	51.6	55.9	59.3	61.5
PHD intake	100 g/day															
Intake adjusted for Energy requirements	49.2	55.2	58	61.2	64.4	68	71.6	76	80	84.4	90	96.8	104	109.6	114.8	118

VIT.B6 requirements (mg/day)	0.6	0.6	0.7	0.7	0.7	1	1	1	1	1.3	1.3	1.3	1.3	1.6	1.6	1.6
PHD intake	2.4 mg/day															

Intake adjusted for Energy requirements	1.2	1.3	1.4	1.5	1.5	1.6	1.7	1.8	1.9	2.0	2.2	2.3	2.5	2.6	2.8	2.8

Folate requirements (µg/day)	120	120	140	140	140	200	200	200	200	270	270	270	270	330	330	330
PHD intake	449 µg/day															
Intake adjusted for Energy requirements	221	248	260	275	289	305	321	341	359	379	404	435	467	492	515	530

VIT.B12 requirements (µg/day)	1.4	1.4	1.7	1.7	1.7	2.5	2.5	2.5	2.5	3.4	3.4	3.4	3.4	4.0	4.0	4.0
PHD intake	4.6 µg/day															
Intake adjusted for Energy requirements	2.3	2.5	2.7	2.8	3.0	3.1	3.3	3.5	3.7	3.9	4.1	4.5	4.8	5.0	5.3	5.4

VIT.C requirements (mg/day)	35	35	45	45	45	60	60	60	60	90	90	90	90	105	105	105
PHD intake	149 mg/day															
Intake adjusted for Energy requirements	73	82	86	91	96	101	107	113	119	126	134	144	155	163	171	176

VIT.A requirements (µg RE/day)	250	250	300	300	300	400	400	400	400	600	600	600	600	750	750	750
PHD intake	1025 µg RE/day															

Intake adjusted for Energy requirements	504	566	595	627	660	697	734	779	820	865	923	992	1066	1123	1177	1210

VIT.E requirements (mg alpha-TE/day)	5	5	6	6	6	8	8	8	8	11	11	11	11	13	13	13
PHD intake	36 alpha-TE mg/day															
Intake adjusted for Energy requirements	18	20	21	22	23	24	26	27	29	30	32	35	37	39	41	42

VIT.D requirements (µg/day)	15	15	15	15	15	15	15	15	15	15	15	15	15	15	15	15
PHD intake	1.5 µg/day															
Intake adjusted for Energy requirements	0.7	0.8	0.9	0.9	1.0	1.0	1.1	1.2	1.2	1.3	1.4	1.5	1.6	1.7	1.7	1.8

Sodium requirements (g/day)	0.5	0.5	0.6	0.6	0.6	1	1	1	1	1.5	1.5	1.5	1.5	1.5	1.5	1.5
PHD intake	0.8 g/day															
Intake adjusted for Energy requirements	0.4	0.4	0.5	0.5	0.5	0.5	0.6	0.6	0.6	0.7	0.7	0.8	0.8	0.9	0.9	0.9

Potassium requirements (mg/day)	1500	1500	1900	1900	1900	2700	2700	2700	2700	4500	4500	4500	4500	4500	4500	4500

PHD intake	3289 mg/day															
Intake adjusted for Energy requirements	1618	1816	1908	2013	2118	2237	2355	2500	2631	2776	2960	3184	3421	3605	3776	3881

Magnesium requirements (mg/day)	120	120	150	150	150	220	220	220	220	290	290	290	290	380	380	380
PHD intake	444 mg/day															
Intake adjusted for Energy requirements	218	245	258	272	286	302	318	337	355	375	400	430	462	487	510	524

Calcium requirements (mg/day)	510	510	900	900	900	1040	1040	1040	1040	1150	1150	1150	1150	1150	1150	1150
PHD intake	1004 mg/day															
Intake adjusted for Energy requirements	494	554	582	614	647	683	719	763	803	847	904	972	1044	1100	1153	1185

Phosphorus requirements (mg/day)	295	295	520	520	520	600	600	600	600	660	660	660	660	660	660	660
PHD intake	1847 mg/day															
Intake adjusted for Energy requirements	909	1020	1071	1130	1189	1256	1322	1404	1478	1559	1662	1788	1921	2024	2120	2179

Iron requirements (mg/day)	8	8	11	11	11	13	13	13	13	10	10	10	10	13	13	13
PHD intake	15.5 mg/day															
Intake adjusted for Energy requirements	8	9	9	9	10	11	11	12	12	13	14	15	16	17	18	18

Zinc requirements (mg/day)	5	5	6	6	6	8	8	8	8	12	12	12	12	12	12	12
PHD intake	12.4 mg/day															
Intake adjusted for Energy requirements	6	7	7	8	8	8	9	9	10	10	11	12	13	14	14	15

Iodine requirements (mg/day)	100	100	100	100	100	100	100	100	100	130	130	130	130	130	130	130
PHD intake	61 µg/day															
Intake adjusted for Energy requirements	30	34	36	38	40	42	44	47	49	52	55	59	64	67	70	72

Selenium requirements (mg/day)	20	20	25	25	25	35	35	35	35	50	50	50	50	55	55	55
PHD intake	36 µg/day															

Intake adjusted for Energy requirements	18	20	21	22	23	24	26	27	29	30	32	35	37	39	41	42

Table S20: Comparison with Reference Intake Levels for the Italian Population (LARN, 2024) for Females, aged 2-17 years.

YEARS (AGE)	2-3	3-4	4-5	5-6	6-7	7-8	8-9	9-10	10-11	11-12	12-13	13-14	14-15	15-16	16-17	17-18
PHD intake	2500 kcal/day															
Energy requirements	1150	1280	1350	1410	1480	1560	1650	1770	1850	1960	2060	2150	2230	2280	2310	2340

Dietary fiber requirements (g/day)	10	11	11	12	12	13	14	15	16	16	17	18	19	19	19	20
PHD intake	35 g/day															
Intake adjusted for Energy requirements	16	18	19	20	21	22	23	25	26	27	29	30	31	32	32	33

Proteins requirements (g/day)	13.3	14.6	16	17.6	20.4	23.1	26.2	29.7	33.3	37.5	41.9	45.5	48.4	49.6	50.3	50.7
PHD intake	100 g/day															
Intake adjusted for Energy requirements	46	51.2	54	56.4	59.2	62.4	66	70.8	74	78.4	82.4	86	89.2	91.2	92.4	93.6

VIT.B6 requirements (mg/day)	0.6	0.6	0.7	0.7	0.7	1	1	1	1	1.3	1.3	1.3	1.3	1.4	1.4	1.4
PHD intake	2.4 mg/day															

Intake adjusted for Energy requirements	1.10	1.23	1.30	1.35	1.42	1.50	1.58	1.70	1.78	1.88	1.98	2.06	2.14	2.19	2.22	2.25

Folate requirements (µg/day)	120	120	140	140	140	200	200	200	200	270	270	270	270	330	330	330
PHD intake	449 µg/day															
Intake adjusted for Energy requirements	207	230	242	253	266	280	296	318	332	352	370	386	401	409	415	420

VIT.B12 requirements (µg/day)	1.4	1.4	1.7	1.7	1.7	2.5	2.5	2.5	2.5	3.4	3.4	3.4	3.4	4.0	4.0	4.0
PHD intake	4.6 µg/day															
Intake adjusted for Energy requirements	2.1	2.4	2.5	2.6	2.7	2.9	3.0	3.3	3.4	3.6	3.8	4.0	4.1	4.2	4.3	4.3

VIT.C requirements (mg/day)	35	35	45	45	45	60	60	60	60	80	80	80	80	85	85	85
PHD intake	149 mg/day															
Intake adjusted for Energy requirements	69	76	80	84	88	93	98	105	110	117	123	128	133	136	138	139

VIT.A requirements (µg RE/day)	250	250	300	300	300	400	400	400	400	600	600	600	600	650	650	650

PHD intake	1025 µg RE/day															
Intake adjusted for Energy requirements	472	525	554	578	607	640	677	726	759	804	845	882	914	935	947	959

VIT.E requirements (mg alpha-TE/day)	5	5	6	6	6	8	8	8	8	11	11	11	11	12	12	12
PHD intake	36 alpha-TE mg/day															
Intake adjusted for Energy requirements	17	18	19	20	21	22	24	25	27	28	30	31	32	33	33	34

VIT.D requirements (µg/day)	15	15	15	15	15	15	15	15	15	15	15	15	15	15	15	15
PHD intake	1.5 µg/day															
Intake adjusted for Energy requirements	0.7	0.8	0.8	0.9	0.9	0.9	1.0	1.1	1.1	1.2	1.3	1.3	1.4	1.4	1.4	1.4

Sodium requirements (g/day)	0.5	0.5	0.6	0.6	0.6	1	1	1	1	1.5	1.5	1.5	1.5	1.5	1.5	1.5
PHD intake	0.8 g/day															
Intake adjusted for Energy requirements	0.4	0.4	0.4	0.5	0.5	0.5	0.5	0.6	0.6	0.6	0.7	0.7	0.7	0.7	0.7	0.7

Potassium requirements (mg/day)	1500	1500	1900	1900	1900	2700	2700	2700	2700	4500	4500	4500	4500	4500	4500	4500
PHD intake	3289 mg/day															
Intake adjusted for Energy requirements	1513	1684	1776	1855	1947	2052	2171	2329	2434	2579	2710	2829	2934	3000	3039	3079

Magnesium requirements (mg/day)	120	120	150	150	150	220	220	220	220	290	290	290	290	310	310	310
PHD intake	444 mg/day															
Intake adjusted for Energy requirements	204	227	240	250	263	277	293	314	329	348	366	382	396	405	410	416

Calcium requirements (mg/day)	510	510	900	900	900	1040	1040	1040	1040	1150	1150	1150	1150	1150	1150	1150
PHD intake	1004 mg/day															
Intake adjusted for Energy requirements	462	514	542	566	594	626	663	711	743	787	827	863	896	916	928	940

Phosphorus requirements (mg/day)	295	295	520	520	520	600	600	600	600	660	660	660	660	660	660	660
PHD intake	1847 mg/day															
Intake adjusted for Energy requirements	850	946	997	1042	1093	1153	1219	1308	1367	1448	1522	1588	1648	1684	1707	1729

Iron requirements (mg/day)	8	8	11	11	11	13	13	13	13	10	10	10	10	18	18	18
PHD intake	15.5 mg/day															
Intake adjusted for Energy requirements	7	8	8	9	9	10	10	11	11	12	13	13	14	14	14	15

Zinc requirements (mg/day)	5	5	6	6	6	8	8	8	8	9	9	9	9	9	9	9
PHD intake	12.4 mg/day															
Intake adjusted for Energy requirements	6	6	7	7	7	8	8	9	9	10	10	11	11	11	11	12

Iodine requirements (mg/day)	100	100	100	100	100	100	100	100	100	130	130	130	130	130	130	130
PHD intake	61 µg/day															
Intake adjusted for Energy requirements	28	31	33	35	36	38	41	43	45	48	51	53	55	56	57	57

Selenium requirements (mg/day)	20	20	25	25	25	35	35	35	35	50	50	50	50	55	55	55
PHD intake	36 µg/day															

Intake adjusted for Energy requirements	17	18	19	20	21	22	24	25	27	28	30	31	32	33	33	34

Table S21: Comparison with Reference Intake Levels for the European Population (EFSA) for Males, aged 2-17 years.

YEARS (AGE)	2-3	3-4	4-5	5-6	6-7	7-8	8-9	9-10	10-11	11-12	12-13	13-14	14-15	15-16	16-17	17-18
PHD intake	2500 kcal/day															
Energy requirements	1027	1170	1433	1529	1600	1720	1815	1935	1935	2030	2174	2341	2508	2699	2842	2938

Dietary fiber requirements (g/day)	10	10	14	14	14	16	16	16	16	19	19	19	19	21	21	21
PHD intake	35 g/day															
Intake adjusted for Energy requirements	14	16	20	21	22	24	25	27	27	28	30	33	35	38	40	41

Proteins requirements (g/day)	12	13	15	16	19	22	25	28	31	34	37	42	47	52	56	58
PHD intake	100 g/day															
Intake adjusted for Energy requirements	41	47	57	61	64	69	73	77	77	81	87	94	100	108	114	118

VIT.B6 requirements (mg/day)	0.6	0.6	0.7	0.7	0.7	1	1	1	1	1.4	1.4	1.4	1.4	1.8	1.8	1.8
PHD intake	2.4 mg/day															

Intake adjusted for Energy requirements	1.0	1.1	1.4	1.5	1.5	1.7	1.7	1.9	1.9	1.9	2.1	2.2	2.4	2.6	2.7	2.8

Folate requirements (µg/day)	120	120	140	140	140	200	200	200	200	270	270	270	270	330	330	330
PHD intake	449 µg/day															
Intake adjusted for Energy requirements	184	210	257	275	287	309	326	348	348	365	390	420	450	485	510	528

VIT.B12 requirements (µg/day)	1.4	1.4	1.7	1.7	1.7	2.5	2.5	2.5	2.5	3.5	3.5	3.5	3.5	4.0	4.0	4.0
PHD intake	4.6 µg/day															
Intake adjusted for Energy requirements	1.9	2.2	2.6	2.8	2.9	3.2	3.3	3.6	3.6	3.7	4.0	4.3	4.6	5.0	5.2	5.4

VIT.C requirements (mg/day)	20	20	30	30	30	45	45	45	45	70	70	70	70	100	100	100
PHD intake	149 mg/day															
Intake adjusted for Energy requirements	61	70	85	91	95	103	108	115	115	121	130	140	149	161	169	175

VIT.A requirements (µg RE/day)	250	250	300	300	300	400	400	400	400	600	600	600	600	750	750	750

PHD intake	1025 µg RE/day															
Intake adjusted for Energy requirements	421	480	588	627	656	705	744	793	793	832	891	960	1028	1107	1165	1205

VIT.E requirements (mg alpha-TE/day)	6	6	9	9	9	9	9	9	9	13	13	13	13	13	13	13
PHD intake	36 alpha-TE mg/day															
Intake adjusted for Energy requirements	15	17	21	22	23	25	26	28	28	29	31	34	36	39	41	42

VIT.D requirements (µg/day)	15	15	15	15	15	15	15	15	15	15	15	15	15	15	15	15
PHD intake	1.5 µg/day															
Intake adjusted for Energy requirements	0.6	0.7	0.9	0.9	1.0	1.0	1.1	1.2	1.2	1.2	1.3	1.4	1.5	1.6	1.7	1.8

Sodium requirements (g/day)	1.1	1.1	1.3	1.3	1.3	1.7	1.7	1.7	1.7	2	2	2	2	2	2	2
PHD intake	0.8 g/day															
Intake adjusted for Energy requirements	0.3	0.4	0.5	0.5	0.5	0.6	0.6	0.6	0.6	0.6	0.7	0.7	0.8	0.9	0.9	0.9

Potassium requirements (mg/day)	800	800	1100	1100	1100	1800	1800	1800	1800	2700	2700	2700	2700	3500	3500	3500
PHD intake	3289 mg/day															
Intake adjusted for Energy requirements	1351	1539	1885	2012	2105	2263	2388	2546	2546	2671	2860	3080	3300	3551	3739	3865

Magnesium requirements (mg/day)	170	170	230	230	230	230	230	230	300	300	300	300	300	300	300	300
PHD intake	444 mg/day															
Intake adjusted for Energy requirements	182	208	255	272	284	305	322	344	344	361	386	416	445	479	505	522

Calcium requirements (mg/day)	388	388	681	681	681	672	672	672	672	944	944	944	944	965	965	965
PHD intake	1004 mg/day															
Intake adjusted for Energy requirements	412	470	575	614	643	691	729	777	777	815	873	940	1007	1084	1141	1180

Phosphorus requirements (mg/day)	250	250	440	440	440	440	440	440	440	640	640	640	640	640	640	640
PHD intake	1847 mg/day															
Intake adjusted for Energy requirements	759	864	1059	1130	1182	1271	1341	1430	1430	1500	1606	1730	1853	1994	2100	2171

Iron requirements (mg/day)	7	7	7	7	7	11	11	11	11	11	11	11	11	11	11	11
PHD intake	15.5 mg/day															
Intake adjusted for Energy requirements	6	7	9	9	10	11	11	12	12	13	13	15	16	17	18	18

Zinc requirements (mg/day)	3.6	4.6	4.6	4.6	4.6	6.2	6.2	6.2	6.2	8.8	8.8	8.8	8.8	11.8	11.8	11.8
PHD intake	12.4 mg/day															
Intake adjusted for Energy requirements	5	6	7	8	8	9	9	10	10	10	11	12	12	13	14	15

Iodine requirements (mg/day)	90	90	90	90	90	90	90	90	90	120	120	120	120	130	130	130
PHD intake	61 µg/day															
Intake adjusted for Energy requirements	25	29	35	38	39	42	45	48	48	50	53	57	62	66	70	72

Selenium requirements (mg/day)	15	15	20	20	20	35	35	35	35	55	55	55	55	70	70	70
PHD intake	36 µg/day															

Intake adjusted for Energy requirements	15	17	21	22	23	25	26	28	28	29	31	34	36	39	41	42

Table S22: Comparison with Reference Intake Levels for the European Population (EFSA) for Females, aged 2-17 years.

YEARS (AGE)	2-3	3-4	4-5	5-6	6-7	7-8	8-9	9-10	10-11	11-12	12-13	13-14	14-15	15-16	16-17	17-18
PHD intake	2500 kcal/day															
Energy requirements	955	1099	1338	1409	1505	1600	1696	1791	1815	1911	2006	2102	2174	2221	2269	2269

Dietary fiber requirements (g/day)	10	10	14	14	14	16	16	16	16	19	19	19	19	21	21	21
PHD intake	35 g/day															
Intake adjusted for Energy requirements	13	15	19	20	21	22	24	25	25	27	28	29	30	31	32	32

Proteins requirements (g/day)	12	13	14	16	19	22	25	28	31	34	38	42	45	46	47	48
PHD intake	100 g/day															
Intake adjusted for Energy requirements	38	44	54	56	60	64	68	72	73	76	80	84	87	89	91	91

VIT.B6 requirements (mg/day)	0.6	0.6	0.7	0.7	0.7	1	1	1	1	1.4	1.4	1.4	1.4	1.6	1.6	1.6
PHD intake	2.4 mg/day															

Intake adjusted for Energy requirements	0.9	1.1	1.3	1.4	1.4	1.5	1.6	1.7	1.7	1.8	1.9	2.0	2.1	2.1	2.2	2.2

Folate requirements (µg/day)	120	120	140	140	140	200	200	200	200	270	270	270	270	330	330	330
PHD intake	449 µg/day															
Intake adjusted for Energy requirements	172	197	240	253	270	287	305	322	326	343	360	378	390	399	408	408

VIT.B12 requirements (µg/day)	1.4	1.4	1.7	1.7	1.7	2.5	2.5	2.5	2.5	3.5	3.5	3.5	3.5	4.0	4.0	4.0
PHD intake	4.6 µg/day															
Intake adjusted for Energy requirements	1.8	2.0	2.5	2.6	2.8	2.9	3.1	3.3	3.3	3.5	3.7	3.9	4.0	4.1	4.2	4.2

VIT.C requirements (mg/day)	20	20	30	30	30	45	45	45	45	70	70	70	70	90	90	90
PHD intake	149 mg/day															
Intake adjusted for Energy requirements	57	66	80	84	90	95	101	107	108	114	120	125	130	132	135	135

VIT.A requirements (µg RE/day)	250	250	300	300	300	400	400	400	400	600	600	600	600	650	650	650

PHD intake	1025 µg RE/day															
Intake adjusted for Energy requirements	392	451	549	578	617	656	695	734	744	784	822	862	891	911	930	930

VIT.E requirements (mg alpha-TE/day)	6	6	9	9	9	9	9	9	9	11	11	11	11	11	11	11
PHD intake	36 alpha-TE mg/day															
Intake adjusted for Energy requirements	14	16	19	20	22	23	24	26	26	28	29	30	31	32	33	33

VIT.D requirements (µg/day)	15	15	15	15	15	15	15	15	15	15	15	15	15	15	15	15
PHD intake	1.5 µg/day															
Intake adjusted for Energy requirements	0.6	0.7	0.8	0.9	0.9	1.0	1.0	1.1	1.1	1.2	1.2	1.3	1.3	1.4	1.4	1.4

Sodium requirements (g/day)	1.1	1.1	1.3	1.3	1.3	1.7	1.7	1.7	1.7	2	2	2	2	2	2	2
PHD intake	0.8 g/day															
Intake adjusted for Energy requirements	0.3	0.4	0.4	0.5	0.5	0.5	0.5	0.6	0.6	0.6	0.6	0.7	0.7	0.7	0.7	0.7

Potassium requirements (mg/day)	800	800	1100	1100	1100	1800	1800	1800	1800	2700	2700	2700	2700	3500	3500	3500
PHD intake	3289 mg/day															
Intake adjusted for Energy requirements	1256	1446	1760	1854	1980	2105	2231	2356	2388	2514	2639	2765	2860	2922	2985	2985

Magnesium requirements (mg/day)	170	170	230	230	230	230	230	230	250	250	250	250	250	250	250	250
PHD intake	444 mg/day															
Intake adjusted for Energy requirements	170	195	238	250	267	284	301	318	322	339	356	373	386	394	403	403

Calcium requirements (mg/day)	388	388	681	681	681	672	672	672	672	944	944	944	944	965	965	965
PHD intake	1004 mg/day															
Intake adjusted for Energy requirements	384	441	537	566	604	643	681	719	729	767	806	844	873	892	911	911

Phosphorus requirements (mg/day)	250	250	440	440	440	440	440	440	440	640	640	640	640	640	640	640
PHD intake	1847 mg/day															
Intake adjusted for Energy requirements	706	812	989	1041	1112	1182	1253	1323	1341	1412	1482	1553	1606	1641	1676	1676

Iron requirements (mg/day)	7	7	7	7	7	11	11	11	11	11	13	13	13	13	13	13
PHD intake	15.5 mg/day															
Intake adjusted for Energy requirements	6	7	8	9	9	10	11	11	11	12	12	13	13	14	14	14

Zinc requirements (mg/day)	3.6	3.6	4.6	4.6	4.6	6.2	6.2	6.2	6.2	8.9	8.9	8.9	8.9	9.9	9.9	9.9
PHD intake	12.4 mg/day															
Intake adjusted for Energy requirements	5	5	7	7	7	8	8	9	9	9	10	10	11	11	11	11

Iodine requirements (mg/day)	90	90	90	90	90	90	90	90	90	120	120	120	120	130	130	130
PHD intake	61 µg/day															
Intake adjusted for Energy requirements	23	27	33	35	37	39	42	44	45	47	49	52	53	55	56	56

Selenium requirements (mg/day)	15	15	20	20	20	35	35	35	35	55	55	55	55	70	70	70
PHD intake	36 µg/day															

Intake adjusted for Energy requirements	14	16	19	20	22	23	24	26	26	28	29	30	31	32	33	33

Table S23: Comparison with Reference Intake Levels for the Global Population (WHO/FAO) for Males, aged 2-17 years

YEARS (AGE)	2-3	3-4	4-5	5-6	6-7	7-8	8-9	9-10	10-11	11-12	12-13	13-14	14-15	15-16	16-17	17-18
PHD intake	2500 kcal/day															
Energy requirements	1125	1250	1350	1475	1575	1700	1825	1975	2150	2350	2550	2775	3000	3175	3325	3400
Dietary fiber requirements (g/day)	15	15	15	15	21	21	21	21	25	25	25	25	25	25	25	25
PHD intake	35 g/day															
Intake adjusted for Energy requirements	16	18	19	21	22	24	26	28	30	33	36	39	42	44	47	48
Proteins requirements (g/day)	12.9	13.8	14.9	16.5	19.3	21.9	24.6	27.2	29.9	33	37.4	42.7	48.3	51.2	54.9	57.5
PHD intake	100 g/day															
Intake adjusted for Energy requirements	45	50	54	59	63	68	73	79	86	94	102	111	120	127	133	136
VIT.B6 requirements (mg/day)	0.5	0.5	0.6	0.6	0.6	1	1	1	1.3	1.3	1.3	1.3	1.3	1.3	1.3	1.3
PHD intake	2.4 mg/day															

Intake adjusted for Energy requirements	1.1	1.2	1.3	1.4	1.5	1.6	1.8	1.9	2.1	2.3	2.4	2.7	2.9	3.0	3.2	3.3

Folate requirements (µg/day)	160	160	200	200	200	300	300	300	400	400	400	400	400	400	400	400
PHD intake	449 µg/day															
Intake adjusted for Energy requirements	202	225	242	265	283	305	328	355	386	422	458	498	539	570	597	611

VIT.B12 requirements (µg/day)	0.9	0.9	1.2	1.2	1.2	1.8	1.8	1.8	2.4	2.4	2.4	2.4	2.4	2.4	2.4	2.4
PHD intake	4.6 µg/day															
Intake adjusted for Energy requirements	2.1	2.3	2.5	2.7	2.9	3.1	3.4	3.6	4.0	4.3	4.7	5.1	5.5	5.8	6.1	6.3

VIT.C requirements (mg/day)	30	30	30	30	30	30	30	30	30	30	30	30	30	30	30	30
PHD intake	149 mg/day															
Intake adjusted for Energy requirements	67	75	80	88	94	101	109	118	128	140	152	165	179	189	198	203

VIT.A requirements (µg RE/day)	400	400	450	450	450	500	500	500	600	600	600	600	600	600	600	600

PHD intake	1025 µg RE/day															
Intake adjusted for Energy requirements	461	513	554	605	646	697	748	810	882	964	1046	1138	1230	1302	1363	1394

VIT.E requirements (mg alpha-TE/day)	5	5	5	5	5	7	7	7	10	10	10	10	10	10	10	10
PHD intake	36 alpha-TE mg/day															
Intake adjusted for Energy requirements	16	18	19	21	23	24	26	28	31	34	37	40	43	46	48	49

VIT.D requirements (µg/day)	5	5	5	5	5	5	5	5	5	5	5	5	5	5	5	5
PHD intake	1.5 µg/day															
Intake adjusted for Energy requirements	0.7	0.8	0.8	0.9	1.0	1.0	1.1	1.2	1.3	1.4	1.6	1.7	1.8	1.9	2.0	2.1

Magnesium requirements (mg/day)	60	60	73	73	73	100	100	100	250	250	250	250	250	250	250	250
PHD intake	444 mg/day															
Intake adjusted for Energy requirements	200	222	240	262	280	302	324	351	382	417	453	493	533	564	591	604

Calcium requirements (mg/day)	500	500	600	600	600	700	700	700	1300	1300	1300	1300	1300	1300	1300	1300
PHD intake	1004 mg/day															
Intake adjusted for Energy requirements	452	502	542	592	633	683	733	793	863	944	1024	1114	1205	1275	1335	1365

Iron requirements (mg/day)	5	5	5	5	5	7	7	7	12	12	12	12	12	16	16	16
PHD intake	15.5 mg/day															
Intake adjusted for Energy requirements	7	8	8	9	10	11	11	12	13	15	16	17	19	20	21	21

Zinc requirements (mg/day)	4.1	4.1	5.1	5.1	5.1	5.6	5.6	5.6	9.7	9.7	9.7	9.7	9.7	9.7	9.7	9.7
PHD intake	12.4 mg/day															
Intake adjusted for Energy requirements	6	6	7	7	8	8	9	10	11	12	13	14	15	16	16	17

Iodine requirements (mg/day)	75	75	110	110	110	100	100	100	135	135	110	110	110	110	110	110
PHD intake	61 µg/day															
Intake adjusted for Energy requirements	28	31	33	36	39	42	45	49	53	58	63	68	74	78	82	84

Selenium requirements (mg/day)	17	17	21	21	21	21	21	21	34	34	34	34	34	34	34	34
PHD intake	36 µg/day															
Intake adjusted for Energy requirements	16	18	19	21	23	24	26	28	31	34	37	40	43	46	48	49

Table S24: Comparison with Reference Intake Levels for the Global Population (WHO/FAO) for Females, aged 2-17 years

YEARS (AGE)	2-3	3-4	4-5	5-6	6-7	7-8	8-9	9-10	10-11	11-12	12-13	13-14	14-15	15-16	16-17	17-18
PHD intake	2500 kcal/day															
Energy requirements	1125	1250	1350	1475	1575	1700	1825	1975	2150	2350	2550	2775	3000	3175	3325	3400

Dietary fiber requirements (g/day)	15	15	15	15	21	21	21	21	25	25	25	25	25	25	25	25
PHD intake	35 g/day															
Intake adjusted for Energy requirements	16	18	19	21	22	24	26	28	30	33	36	39	42	44	47	48

Proteins requirements (g/day)	12.3	13.5	14.8	16.2	18.9	21.5	24.4	27.6	30.9	34.4	38.8	42.6	45.8	45.3	46.5	47.3
PHD intake	100 g/day															
Intake adjusted for Energy requirements	45	50	54	59	63	68	73	79	86	94	102	111	120	127	133	136

VIT.B6 requirements (mg/day)	0.5	0.5	0.6	0.6	0.6	1	1	1	1.2	1.2	1.2	1.2	1.2	1.2	1.2	1.2
PHD intake	2.4 mg/day															

Intake adjusted for Energy requirements	1.1	1.2	1.3	1.4	1.5	1.6	1.8	1.9	2.1	2.3	2.4	2.7	2.9	3.0	3.2	3.3

Folate requirements (µg/day)	160	160	200	200	200	300	300	300	400	400	400	400	400	400	400	400
PHD intake	449 µg/day															
Intake adjusted for Energy requirements	202	225	242	265	283	305	328	355	386	422	458	498	539	570	597	611

VIT.B12 requirements (µg/day)	0.9	0.9	1.2	1.2	1.2	1.8	1.8	1.8	2.4	2.4	2.4	2.4	2.4	2.4	2.4	2.4
PHD intake	4.6 µg/day															
Intake adjusted for Energy requirements	2.1	2.3	2.5	2.7	2.9	3.1	3.4	3.6	4.0	4.3	4.7	5.1	5.5	5.8	6.1	6.3

VIT.C requirements (mg/day)	30	30	30	30	30	30	30	30	30	30	30	30	30	30	30	30
PHD intake	149 mg/day															
Intake adjusted for Energy requirements	67	75	80	88	94	101	109	118	128	140	152	165	179	189	198	203

VIT.A requirements (µg RE/day)	400	400	450	450	450	500	500	500	600	600	600	600	600	600	600	600

PHD intake	1025 µg RE/day															
Intake adjusted for Energy requirements	461	513	554	605	646	697	748	810	882	964	1046	1138	1230	1302	1363	1394

VIT.E requirements (mg alpha-TE/day)	5	5	5	5	5	7	7	7	7.5	7.5	7.5	7.5	7.5	7.5	7.5	7.5
PHD intake	36 alpha-TE mg/day															
Intake adjusted for Energy requirements	16	18	19	21	23	24	26	28	31	34	37	40	43	46	48	49

VIT.D requirements (µg/day)	5	5	5	5	5	5	5	5	5	5	5	5	5	5	5	5
PHD intake	1.5 µg/day															
Intake adjusted for Energy requirements	0.7	0.8	0.8	0.9	1.0	1.0	1.1	1.2	1.3	1.4	1.6	1.7	1.8	1.9	2.0	2.1

Magnesium requirements (mg/day)	60	60	73	73	73	100	100	100	230	230	230	230	230	230	230	230
PHD intake	444 mg/day															
Intake adjusted for Energy requirements	200	222	240	262	280	302	324	351	382	417	453	493	533	564	591	604

Calcium requirements (mg/day)	500	500	600	600	600	700	700	700	1300	1300	1300	1300	1300	1300	1300	1300
PHD intake	1004 mg/day															
Intake adjusted for Energy requirements	452	502	542	592	633	683	733	793	863	944	1024	1114	1205	1275	1335	1365

Iron requirements (mg/day)	5	5	5	5	5	7	7	7	12/28	12/28	12/28	12/28	12/28	26	26	26
PHD intake	15.5 mg/day															
Intake adjusted for Energy requirements	7	8	8	9	10	11	11	12	13	15	16	17	19	20	21	21

Zinc requirements (mg/day)	4.1	4.1	5.1	5.1	5.1	5.6	5.6	5.6	7.8	7.8	7.8	7.8	7.8	7.8	7.8	7.8
PHD intake	12.4 mg/day															
Intake adjusted for Energy requirements	6	6	7	7	8	8	9	10	11	12	13	14	15	16	16	17

Iodine requirements (mg/day)	75	75	110	110	110	100	100	100	140	140	100	100	100	100	100	100
PHD intake	61 µg/day															
Intake adjusted for Energy requirements	28	31	33	36	39	42	45	49	53	58	63	68	74	78	82	84

Selenium requirements (mg/day)	17	17	21	21	21	21	21	21	26	26	26	26	26	26	26	26
PHD intake	36 µg/day															
Intake adjusted for Energy requirements	16	18	19	21	23	24	26	28	31	34	37	40	43	46	48	49

Table S25: Adequacy of PHD in meeting Reference Intake Levels according to LARN, EFSA and WHO/FAO recommendations for females and males, aged 2-17 years

Table S25a. Adequacy of PHD in meeting energy recommendation of females and males, 2 to 17 years, according to LARN, EFSA and FAO/WHO.

YEARS (AGE)	2-3	3-4	4-5	5-6	6-7	7-8	8-9	9-10	10-11	11-12	12-13	13-14	14-15	15-16	16-17	17-18
Females																
LARN	1150	1280	1350	1410	1480	1560	1650	1770	1850	1960	2060	2150	2230	2280	2310	2340
EFSA	955	1099	1338	1409	1505	1600	1696	1791	1815	1911	2006	2102	2174	2221	2269	2269
FAO/WHO	1125	1250	1350	1475	1575	1700	1825	1975	2150	2350	2550	2775	3000	3175	3325	3400
Males																
LARN	1230	1380	1450	1530	1610	1700	1790	1900	2000	2110	2250	2420	2600	2740	2870	2950
EFSA	1027	1170	1433	1529	1600	1720	1815	1935	1935	2030	2174	2341	2508	2699	2842	2938
FAO/WHO	1125	1250	1350	1475	1575	1700	1825	1975	2150	2350	2550	2775	3000	3175	3325	3400

Legend Table S25a. Energy intake is reported as kcal/day. Color coding: green, adequate; yellow, relatively adequate; red: inadequate.

Table S25b. Adequacy of PHD in meeting dietary fiber recommendation of females and males, 2 to 17 years, according to LARN, EFSA and FAO/WHO.

YEARS (AGE)	2-3	3-4	4-5	5-6	6-7	7-8	8-9	9-10	10-11	11-12	12-13	13-14	14-15	15-16	16-17	17-18
Females																
LARN	16	18	19	20	21	22	23	25	26	27	29	30	31	32	32	33
EFSA	13	15	19	20	21	22	24	25	25	27	28	29	30	31	32	32
FAO/WHO	16	18	19	21	22	24	26	28	30	33	36	39	42	44	47	48
Males																
LARN	17	19	20	21	23	24	25	27	28	30	32	34	36	38	40	41
EFSA	14	16	20	21	22	24	25	27	27	28	30	33	35	38	40	41
FAO/WHO	16	18	19	21	22	24	26	28	30	33	36	39	42	44	47	48

Legend Table S25b. Dietary fiber intake is reported as g/day. Color coding: green, adequate; yellow, relatively adequate; red: inadequate.

Table S25c. Adequacy of PHD in meeting protein recommendation of females and males, 2 to 17 years, according to LARN, EFSA and FAO/WHO.

YEARS (AGE)	2-3	3-4	4-5	5-6	6-7	7-8	8-9	9-10	10-11	11-12	12-13	13-14	14-15	15-16	16-17	17-18
Females																
LARN	46.0	51.2	54.0	56.4	59.2	62.4	66.0	70.8	74.0	78.4	82.4	86.0	89.2	91.2	92.4	93.6
EFSA	38.0	44.0	54.0	56.0	60.0	64.0	68.0	72.0	73.0	76.0	80.0	84.0	87.0	89.0	91.0	91.0
FAO/WHO	45.0	50.0	54.0	59.0	63.0	68.0	73.0	79.0	86.0	94.0	102.0	111.0	120.0	127.0	133.0	136.0
Males																
LARN	49.2	55.2	58.0	61.2	64.4	68.0	71.6	76.0	80.0	84.4	90.0	96.8	104.0	109.6	114.8	118.0
EFSA	41.0	47.0	57.0	61.0	64.0	69.0	73.0	77.0	77.0	81.0	87.0	94.0	100.0	108.0	114.0	118.0
FAO/WHO	45.0	50.0	54.0	59.0	63.0	68.0	73.0	79.0	86.0	94.0	102.0	111.0	120.0	127.0	133.0	136.0

Legend Table S25c. Protein intake is reported as g/day. Color coding: green, adequate; yellow, relatively adequate; red: inadequate.

Table S25d. Adequacy of PHD in meeting vitamin B6 recommendation of females and males, 2 to 17 years, according to LARN, EFSA and FAO/WHO.

YEARS (AGE)	2-3	3-4	4-5	5-6	6-7	7-8	8-9	9-10	10-11	11-12	12-13	13-14	14-15	15-16	16-17	17-18
Females																
LARN	1.10	1.23	1.30	1.35	1.42	1.50	1.58	1.70	1.78	1.88	1.98	2.06	2.14	2.19	2.22	2.25
EFSA	0.90	1.10	1.30	1.40	1.40	1.50	1.60	1.70	1.70	1.80	1.90	2.00	2.10	2.10	2.20	2.20
FAO/WHO	1.10	1.20	1.30	1.40	1.50	1.60	1.80	1.90	2.10	2.30	2.40	2.70	2.90	3.00	3.20	3.30
Males																
LARN	1.20	1.30	1.40	1.50	1.50	1.60	1.70	1.80	1.90	2.00	2.20	2.30	2.50	2.60	2.80	2.80
EFSA	1.00	1.10	1.40	1.50	1.50	1.70	1.70	1.90	1.90	1.90	2.10	2.20	2.40	2.60	2.70	2.80
FAO/WHO	1.10	1.20	1.30	1.40	1.50	1.60	1.80	1.90	2.10	2.30	2.40	2.70	2.90	3.00	3.20	3.30

Legend Table S25d. Vitamin B6 intake is reported as g/day. Color coding: green, adequate; yellow, relatively adequate; red: inadequate.

Table S25e. Adequacy of PHD in meeting folate recommendation of females and males, 2 to 17 years, according to LARN, EFSA and FAO/WHO.

YEARS (AGE)	2-3	3-4	4-5	5-6	6-7	7-8	8-9	9-10	10-11	11-12	12-13	13-14	14-15	15-16	16-17	17-18
Females																
LARN	207	230	242	253	266	280	296	318	332	352	370	386	401	409	415	420
EFSA	172	197	240	253	270	287	305	322	326	343	360	378	390	399	408	408
FAO/WHO	202	225	242	265	283	305	328	355	386	422	458	498	539	570	597	611
Males																
LARN	221	248	260	275	289	305	321	341	359	379	404	435	467	492	515	530
EFSA	184	210	257	275	287	309	326	348	348	365	390	420	450	485	510	528
FAO/WHO	202	225	242	265	283	305	328	355	386	422	458	498	539	570	597	611

Legend Table S25e. Folate intake is reported as g/day. Color coding: green, adequate; yellow, relatively adequate; red: inadequate.

Table S25f. Adequacy of PHD in meeting Vitamin B12 recommendation of females and males, 2 to 17 years, according to LARN, EFSA and FAO/WHO.

YEARS (AGE)	2-3	3-4	4-5	5-6	6-7	7-8	8-9	9-10	10-11	11-12	12-13	13-14	14-15	15-16	16-17	17-18
Females																
LARN	2.1	2.4	2.5	2.6	2.7	2.9	3.0	3.3	3.4	3.6	3.8	4.0	4.1	4.2	4.3	4.3
EFSA	1.8	2.0	2.5	2.6	2.8	2.9	3.1	3.3	3.3	3.5	3.7	3.9	4.0	4.1	4.2	4.2
FAO/WHO	2.1	2.3	2.5	2.7	2.9	3.1	3.4	3.6	4.0	4.3	4.7	5.1	5.5	5.8	6.1	6.3
Males																
LARN	2.3	2.5	2.7	2.8	3.0	3.1	3.3	3.5	3.7	3.9	4.1	4.5	4.8	5.0	5.3	5.4
EFSA	1.9	2.2	2.6	2.8	2.9	3.2	3.3	3.6	3.6	3.7	4.0	4.3	4.6	5.0	5.2	5.4
FAO/WHO	2.1	2.3	2.5	2.7	2.9	3.1	3.4	3.6	4.0	4.3	4.7	5.1	5.5	5.8	6.1	6.3

Legend Table S25f. Vitamin B12 intake is reported as g/day. Color coding: green, adequate; yellow, relatively adequate; red: inadequate.

Table S25g. Adequacy of PHD in meeting Vitamin C recommendation of females and males, 2 to 17 years, according to LARN, EFSA and FAO/WHO.

YEARS (AGE)	2-3	3-4	4-5	5-6	6-7	7-8	8-9	9-10	10-11	11-12	12-13	13-14	14-15	15-16	16-17	17-18
Females																
LARN	69	76	80	84	88	93	98	105	110	117	123	128	133	136	138	139
EFSA	57	66	80	84	90	95	101	107	108	114	120	125	130	132	135	135
FAO/WHO	67	75	80	88	94	101	109	118	128	140	152	165	179	189	198	203
Males																
LARN	73	82	86	91	96	101	107	113	119	126	134	144	155	163	171	176
EFSA	61	70	85	91	95	103	108	115	115	121	130	140	149	161	169	175
FAO/WHO	67	75	80	88	94	101	109	118	128	140	152	165	179	189	198	203

Legend Table S25g. Vitamin C intake is reported as g/day. Color coding: green, adequate; yellow, relatively adequate; red: inadequate.

Table S25h. Adequacy of PHD in meeting Vitamin A recommendation of females and males, 2 to 17 years, according to LARN, EFSA and FAO/WHO.

YEARS (AGE)	2-3	3-4	4-5	5-6	6-7	7-8	8-9	9-10	10-11	11-12	12-13	13-14	14-15	15-16	16-17	17-18
Females																
LARN	472	525	554	578	607	640	677	726	759	804	845	882	914	935	947	959
EFSA	392	451	549	578	617	656	695	734	744	784	822	862	891	911	930	930
FAO/WHO	461	513	554	605	646	697	748	810	882	964	1046	1138	1230	1302	1363	1394
Males																
LARN	504	566	595	627	660	697	734	779	820	865	923	992	1066	1123	1177	1210
EFSA	421	480	588	627	656	705	744	793	793	832	891	960	1028	1107	1165	1205
FAO/WHO	461	513	554	605	646	697	748	810	882	964	1046	1138	1230	1302	1363	1394

Legend Table S25h. Vitamin A intake is reported as g/day. Color coding: green, adequate; yellow, relatively adequate; red: inadequate.

Table S25i. Adequacy of PHD in meeting Vitamin E recommendation of females and males, 2 to 17 years, according to LARN, EFSA and FAO/WHO.

YEARS (AGE)	2-3	3-4	4-5	5-6	6-7	7-8	8-9	9-10	10-11	11-12	12-13	13-14	14-15	15-16	16-17	17-18
Females																
LARN	17	18	19	20	21	22	24	25	27	28	30	31	32	33	33	34
EFSA	14	16	19	20	22	23	24	26	26	28	29	30	31	32	33	33
FAO/WHO	16	18	19	21	23	24	26	28	31	34	37	40	43	46	48	49
Males																
LARN	18	20	21	22	23	24	26	27	29	30	32	35	37	39	41	42
EFSA	15	17	21	22	23	25	26	28	28	29	31	34	36	39	41	42
FAO/WHO	16	18	19	21	23	24	26	28	31	34	37	40	43	46	48	49

Legend Table S25i. Vitamin E intake is reported as g/day. Color coding: green, adequate; yellow, relatively adequate; red: inadequate.

Table S25I. Adequacy of PHD in meeting Vitamin D recommendation of females and males, 2 to 17 years, according to LARN, EFSA and FAO/WHO.

YEARS (AGE)	2-3	3-4	4-5	5-6	6-7	7-8	8-9	9-10	10-11	11-12	12-13	13-14	14-15	15-16	16-17	17-18
Females																
LARN	0.7	0.8	0.8	0.9	0.9	0.9	1.0	1.1	1.1	1.2	1.3	1.3	1.4	1.4	1.4	1.4
EFSA	0.6	0.7	0.8	0.9	0.9	1.0	1.0	1.1	1.1	1.2	1.2	1.3	1.3	1.4	1.4	1.4
FAO/WHO	0.7	0.8	0.8	0.9	1.0	1.0	1.1	1.2	1.3	1.4	1.6	1.7	1.8	1.9	2.0	2.1
Males																
LARN	0.7	0.8	0.9	0.9	1.0	1.0	1.1	1.2	1.2	1.3	1.4	1.5	1.6	1.7	1.7	1.8
EFSA	0.6	0.7	0.9	0.9	1.0	1.0	1.1	1.2	1.2	1.2	1.3	1.4	1.5	1.6	1.7	1.8
FAO/WHO	0.7	0.8	0.8	0.9	1.0	1.0	1.1	1.2	1.3	1.4	1.6	1.7	1.8	1.9	2.0	2.1

Legend Table S25I. Vitamin D intake is reported as g/day. Color coding: green, adequate; yellow, relatively adequate; red: inadequate.

Table S25m. Adequacy of PHD in meeting Sodium recommendation of females and males, 2 to 17 years, according to LARN and EFSA.

YEARS (AGE)	2-3	3-4	4-5	5-6	6-7	7-8	8-9	9-10	10-11	11-12	12-13	13-14	14-15	15-16	16-17	17-18
Females																
LARN	0.4	0.4	0.4	0.5	0.5	0.5	0.5	0.6	0.6	0.6	0.7	0.7	0.7	0.7	0.7	0.7
EFSA	0.3	0.4	0.4	0.5	0.5	0.5	0.5	0.6	0.6	0.6	0.6	0.7	0.7	0.7	0.7	0.7
Males																
LARN	0.4	0.4	0.5	0.5	0.5	0.5	0.6	0.6	0.6	0.7	0.7	0.8	0.8	0.9	0.9	0.9
EFSA	0.3	0.4	0.5	0.5	0.5	0.6	0.6	0.6	0.6	0.6	0.7	0.7	0.8	0.9	0.9	0.9

Legend Table S25m. Sodium intake is reported as g/day. Color coding: green, adequate; yellow, relatively adequate; red: inadequate. WHO has not defined a specific recommendation for this nutrient

Table S25n. Adequacy of PHD in meeting Potassium recommendation of females and males, 2 to 17 years, according to LARN and EFSA.

YEARS (AGE)	2-3	3-4	4-5	5-6	6-7	7-8	8-9	9-10	10-11	11-12	12-13	13-14	14-15	15-16	16-17	17-18
Females																
LARN	1513	1684	1776	1855	1947	2052	2171	2329	2434	2579	2710	2829	2934	3000	3039	3079
EFSA	1256	1446	1760	1854	1980	2105	2231	2356	2388	2514	2639	2765	2860	2922	2985	2985
Males																
LARN	1618	1816	1908	2013	2118	2237	2355	2500	2631	2776	2960	3184	3421	3605	3776	3881
EFSA	1351	1539	1885	2012	2105	2263	2388	2546	2546	2671	2860	3080	3300	3551	3739	3865

Legend Table S25n. Potassium intake is reported as g/day. Color coding: green, adequate; yellow, relatively adequate; red: inadequate. WHO has not defined a specific recommendation for this nutrient

Table S25o. Adequacy of PHD in meeting Magnesium recommendation of females and males, 2 to 17 years, according to LARN, EFSA and FAO/WHO.

YEARS (AGE)	2-3	3-4	4-5	5-6	6-7	7-8	8-9	9-10	10-11	11-12	12-13	13-14	14-15	15-16	16-17	17-18
Females																
LARN	204	227	240	250	263	277	293	314	329	348	366	382	396	405	410	416
EFSA	170	195	238	250	267	284	301	318	322	339	356	373	386	394	403	403
FAO/WHO	200	222	240	262	280	302	324	351	382	417	453	493	533	564	591	604
Males																
LARN	218	245	258	272	286	302	318	337	355	375	400	430	462	487	510	524
EFSA	182	208	255	272	284	305	322	344	344	361	386	416	445	479	505	522
FAO/WHO	200	222	240	262	280	302	324	351	382	417	453	493	533	564	591	604

Legend Table S25o. Magnesium intake is reported as g/day. Color coding: green, adequate; yellow, relatively adequate; red: inadequate.

Table S25p. Adequacy of PHD in meeting Calcium recommendation of females and males, 2 to 17 years, according to LARN, EFSA and FAO/WHO.

YEARS (AGE)	2-3	3-4	4-5	5-6	6-7	7-8	8-9	9-10	10-11	11-12	12-13	13-14	14-15	15-16	16-17	17-18
Females																
LARN	462	514	542	566	594	626	663	711	743	787	827	863	896	916	928	940
EFSA	384	441	537	566	604	643	681	719	729	767	806	844	873	892	911	911
FAO/WHO	452	502	542	592	633	683	733	793	863	944	1024	1114	1205	1275	1335	1365
Males																
LARN	494	554	582	614	647	683	719	763	803	847	904	972	1044	1100	1153	1185
EFSA	412	470	575	614	643	691	729	777	777	815	873	940	1007	1084	1141	1180
FAO/WHO	452	502	542	592	633	683	733	793	863	944	1024	1114	1205	1275	1335	1365

Legend Table S25p. Calcium intake is reported as g/day. Color coding: green, adequate; yellow, relatively adequate; red: inadequate.

Table S25q. Adequacy of PHD in meeting Phosphorus recommendation of females and males, 2 to 17 years, according to LARN and EFSA.

YEARS (AGE)	2-3	3-4	4-5	5-6	6-7	7-8	8-9	9-10	10-11	11-12	12-13	13-14	14-15	15-16	16-17	17-18
Females																
LARN	850	946	997	1042	1093	1153	1219	1308	1367	1448	1522	1588	1648	1684	1707	1729
EFSA	706	812	989	1041	1112	1182	1253	1323	1341	1412	1482	1553	1606	1641	1676	1676
Males																
LARN	909	1020	1071	1130	1189	1256	1322	1404	1478	1559	1662	1788	1921	2024	2120	2179
EFSA	759	864	1059	1130	1182	1271	1341	1430	1430	1500	1606	1730	1853	1994	2100	2171

Legend Table S25q. Phosphorus intake is reported as g/day. Color coding: green, adequate; yellow, relatively adequate; red: inadequate. WHO has not defined a specific recommendation for this nutrient.

Table S25r. Adequacy of PHD in meeting Iron recommendation of females and males, 2 to 17 years, according to LARN, EFSA and FAO/WHO.

YEARS (AGE)	2-3	3-4	4-5	5-6	6-7	7-8	8-9	9-10	10-11	11-12	12-13	13-14	14-15	15-16	16-17	17-18
Females																
LARN	7	8	8	9	9	10	10	11	11	12	13	13	14	14	14	15
EFSA	6	7	8	9	9	10	11	11	11	12	12	13	13	14	14	14
FAO/WHO	7	8	8	9	10	11	11	12	13	15	16	17	19	20	21	21
Males																
LARN	8	9	9	9	10	11	11	12	12	13	14	15	16	17	18	18
EFSA	6	7	9	9	10	11	11	12	12	13	13	15	16	17	18	18
FAO/WHO	7	8	8	9	10	11	11	12	13	15	16	17	19	20	21	21

Legend Table S25r. Iron intake is reported as g/day. Color coding: green, adequate; yellow, relatively adequate; red: inadequate.

Table S25s. Adequacy of PHD in meeting Zinc recommendation of females and males, 2 to 17 years, according to LARN, EFSA and FAO/WHO.

YEARS (AGE)	2-3	3-4	4-5	5-6	6-7	7-8	8-9	9-10	10-11	11-12	12-13	13-14	14-15	15-16	16-17	17-18
Females																
LARN	6	6	7	7	7	8	8	9	9	10	10	11	11	11	11	12
EFSA	5	5	7	7	7	8	8	9	9	9	10	10	11	11	11	11
FAO/WHO	6	6	7	7	8	8	9	10	11	12	13	14	15	16	16	17
Males																
LARN	6	7	7	8	8	8	9	9	10	10	11	12	13	14	14	15
EFSA	5	6	7	8	8	9	9	10	10	10	11	12	12	13	14	15
FAO/WHO	6	6	7	7	8	8	9	10	11	12	13	14	15	16	16	17

Legend Table S25s. Zinc intake is reported as g/day. Color coding: green, adequate; yellow, relatively adequate; red: inadequate.

Table S25t. Adequacy of PHD in meeting Iodine recommendation of females and males, 2 to 17 years, according to LARN, EFSA and FAO/WHO.

YEARS (AGE)	2-3	3-4	4-5	5-6	6-7	7-8	8-9	9-10	10-11	11-12	12-13	13-14	14-15	15-16	16-17	17-18
Females																
LARN	28	31	33	35	36	38	41	43	45	48	51	53	55	56	57	57
EFSA	23	27	33	35	37	39	42	44	45	47	49	52	53	55	56	56
FAO/WHO	28	31	33	36	39	42	45	49	53	58	63	68	74	78	82	84
Males																
LARN	30	34	36	38	40	42	44	47	49	52	55	59	64	67	70	72
EFSA	25	29	35	38	39	42	45	48	48	50	53	57	62	66	70	72
FAO/WHO	28	31	33	36	39	42	45	49	53	58	63	68	74	78	82	84

Legend Table S25t. Iodine intake is reported as g/day. Color coding: green, adequate; yellow, relatively adequate; red: inadequate.

Table S25u. Adequacy of PHD in meeting Selenium recommendation of females and males, 2 to 17 years, according to LARN, EFSA and FAO/WHO.

YEARS (AGE)	2-3	3-4	4-5	5-6	6-7	7-8	8-9	9-10	10-11	11-12	12-13	13-14	14-15	15-16	16-17	17-18
Females																
LARN	17	18	19	20	21	22	24	25	27	28	30	31	32	33	33	34
EFSA	14	16	19	20	22	23	24	26	26	28	29	30	31	32	33	33
FAO/WHO	16	18	19	21	23	24	26	28	31	34	37	40	43	46	48	49
Males																
LARN	18	20	21	22	23	24	26	27	29	30	32	35	37	39	41	42
EFSA	15	17	21	22	23	25	26	28	28	29	31	34	36	39	41	42
FAO/WHO	16	18	19	21	23	24	26	28	31	34	37	40	43	46	48	49

Legend Table S25u. Selenium intake is reported as g/day. Color coding: green, adequate; yellow, relatively adequate; red: inadequate
